# TPPU treatment of burned mice dampens inflammation and generation of bioactive DHET which impairs neutrophil function

**DOI:** 10.1038/s41598-021-96014-2

**Published:** 2021-08-16

**Authors:** Christian B. Bergmann, Bruce D. Hammock, Debin Wan, Falk Gogolla, Holly Goetzman, Charles C. Caldwell, Dorothy M. Supp

**Affiliations:** 1 Division of Research, Department of Surgery, College of Medicine, University of Cincinnati, Cincinnati, OH USA; 2 Department of Entomology, University of California, Davis, CA USA; 3 Institute of Bioinformatics, Medical University of Innsbruck, Innsbruck, Austria; 4 Division of Plastic, Reconstructive and Hand Surgery/Burn Surgery, Department of Surgery, University of Cincinnati College of Medicine, Cincinnati, OH USA; 5 Scientific Staff, Shriners Children’s Ohio, Dayton, OH USA

**Keywords:** Cytokines, Lipidomics, Lipids, Inflammation, Acute inflammation, Applied immunology, Cytokines, Gene regulation in immune cells, Inflammation, Innate immune cells

## Abstract

Oxylipins modulate the behavior of immune cells in inflammation. Soluble epoxide hydrolase (sEH) converts anti-inflammatory epoxyeicosatrienoic acid (EET) to dihydroxyeicosatrienoic acid (DHET). An sEH-inhibitor, TPPU, has been demonstrated to ameliorate lipopolysaccharide (LPS)- and sepsis-induced inflammation via EETs. The immunomodulatory role of DHET is not well characterized. We hypothesized that TPPU dampens inflammation and that sEH-derived DHET alters neutrophil functionality in burn induced inflammation. Outbred mice were treated with vehicle, TPPU or 14,15-DHET and immediately subjected to either sham or dorsal scald 28% total body surface area burn injury. After 6 and 24 h, interleukin 6 (IL-6) serum levels and neutrophil activation were analyzed. For in vitro analyses, bone marrow derived neutrophil functionality and mRNA expression were examined. In vivo, 14,15-DHET and IL-6 serum concentrations were decreased after burn injury with TPPU administration. In vitro, 14,15-DHET impaired neutrophil chemotaxis, acidification, CXCR1/CXCR2 expression and reactive oxygen species (ROS) production, the latter independent from p38MAPK and PI3K signaling. We conclude that TPPU administration decreases DHET post-burn. Furthermore, DHET downregulates key neutrophil immune functions and mRNA expression. Altogether, these data reveal that TPPU not only increases anti-inflammatory and inflammation resolving EET levels, but also prevents potential impairment of neutrophils by DHET in trauma.

## Introduction

The concept that oxylipins can modulate the behavior of immune cells in inflammatory diseases has evolved over the last decades^1–3^. Recently, clinical observational studies in inflammatory diseases and conditions such as sepsis, pneumonia, or surgical trauma assess oxylipin levels with the aim to establish health predictions and alter outcomes based on oxylipin dynamics^4–6^.

In the largely anti-inflammatory branch of the arachidonic acid cascade, epoxy-fatty acids (EpFA) are among the dominant and best studied oxylipins. Four epoxyeicosatrienoic acid (EET) regioisomers are produced from arachidonic acid by cytochrome P-450 (CYP) enzymes by epoxidation of the double bonds^2^. Beside the role of EETs to regulate vascular tone^2,7^, they were found to exert potent anti-inflammatory functions^2,3,8^. EETs are converted to dihydroxyeicosatrienoic acids (DHETs) largely by soluble epoxide hydrolase (sEH)^2^. However, in some tissues and with some EpFA the microsomal epoxide hydrolase (mEH) plays a significant role^9^. Several molecules, e.g. N-[1-(1-oxopropyl)-4-piperidinyl]-N’-[4-(trifluoromethoxy)phenyl)-urea (TPPU), are known to inhibit sEH^10^. In vivo experimental models revealed sEH-inhibitors dampen inflammation. Administering sEH reduced the release of pro-inflammatory cytokines in murine endotoxemia^10,11^, increased survival in a mouse sepsis model^12^, and reduced neuronal death in an intracerebral hemorrhage model^13^. Some studies attributed these effects to the anti-inflammatory properties of EETs^11,12^, which are increased upon inhibition of sEH. What none of these studies examined is whether the effect could be attributed to the eradication of potential immunomodulation by DHET.

Burn trauma models are well suited to examine potential immunomodulatory effects of oxylipins. Burn injury results in a robust systemic activation of both innate and adaptive immunity^14^. Murine burn injury acutely increases relative and absolute numbers of neutrophils^15^, induces acute activation and increase in numbers of Regulatory T cells^15,16^ and impairs CD4 and CD8 T cell function^17^. Neutrophils are a key cellular component for the resolution of the injury as they are first recruited to the site of injury in quantitatively high numbers^18^. Neutrophils’ key functions are the ability to migrate to the site of injury and subsequently clear cell debris and pathogens from injured tissue, by phagocytizing and dissolving them internally by producing reactive oxygen species^18–20^. Recent studies showed that different neutrophil subgroups, distinguished by the expression of intercellular adhesion molecule 1 (ICAM-1, also known as CD54) and CD62L (L-Selectin), differ in their maturation and potency to exert these functions^21–23^.

Previous studies examining modulation of inflammation by oxylipins focused on the effect on macrophages^12,24^, therefore the regulation of neutrophil functionality is not yet well characterized. Based upon this limited literature, we tested whether TPPU would alter systemic inflammation after burn injury and hypothesized that sEH-derived DHETs alter immune function in neutrophils that play key roles in burn induced inflammation.

## Results

### TPPU treatment decreases serum DHET levels and serum IL-6 levels following burn injury, preventing DHET induced neutrophil depression

**Figure 1:**
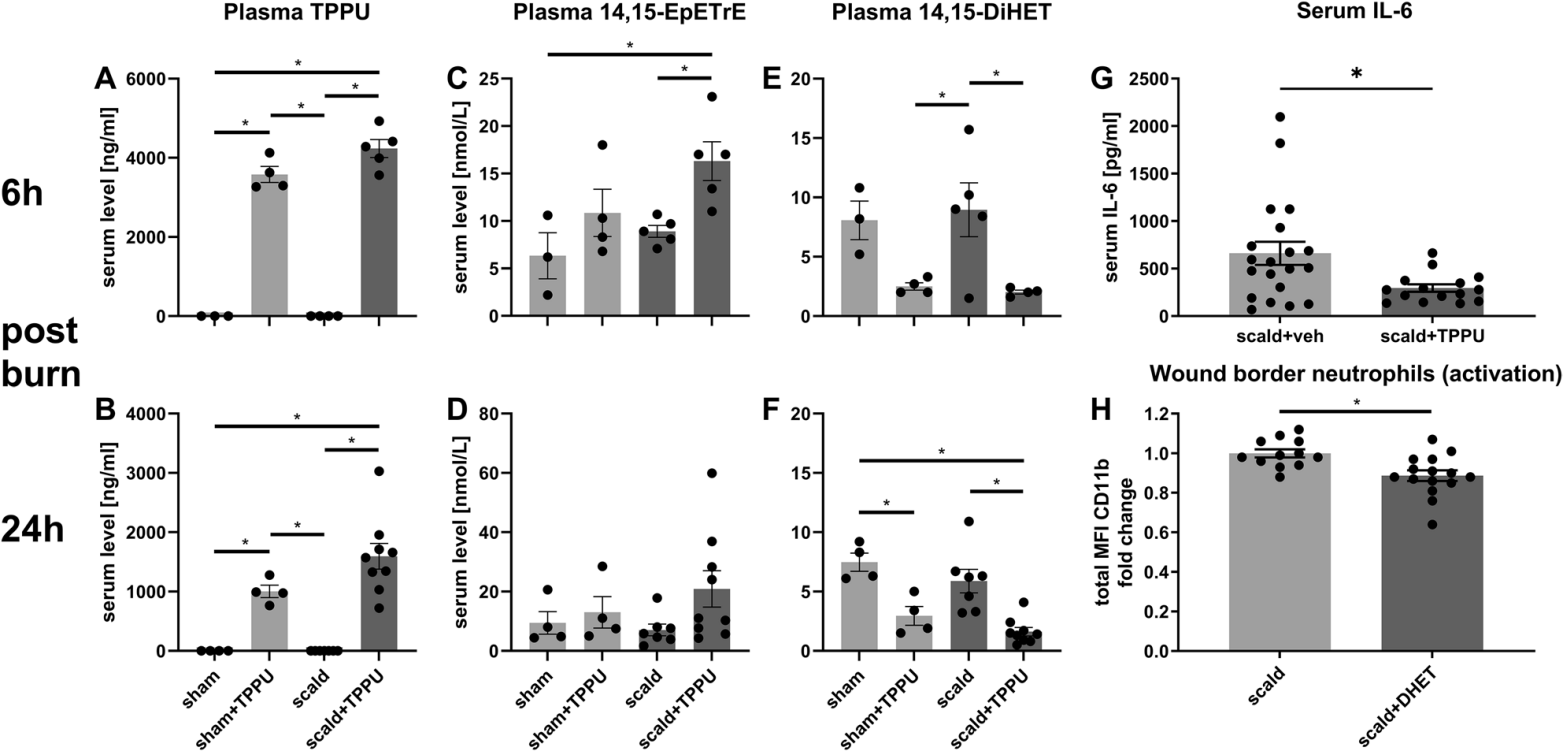
Acutely after burn injury TPPU decreases serum DHET levels and serum IL-6 levels, which prevents DHET induced decrease of neutrophil activation. TPPU increases the serum 14,15-EpETrE levels and decreases serum 14,15-DHET levels after burn injury. TPPU is an inhibitor of soluble epoxide hydrolase (sEH), which converts 14,15-EpETrE into 14,15-DHET. 10 mg/kg body weight TPPU in polyethylene glycol 400 (PEG) or PEG as control were administered intraperitoneally directly post-burn or sham intervention in male CD1 IGS mice (n = 3–5/group). Injury was inflicted by a third degree burn of 28% of total body surface on the back and blood was harvested after 6 or 24 h. Serum levels of TPPU, 14,15 EpETrE and 14,15-DHET were assessed using mass spectroscopy (**A**–**F**). Serum levels of IL-6 were analyzed using Cytometric Bead Array after 6 h (n = 15–20/ group) (**G**). Skin from the burn wound border was harvested and dissected after 24 h to measure the activation of neutrophils after burn injury and intraperitoneal injection of 15 µg/kg body weight 14,15-DHET (n = 12–15/group) or phosphate buffered saline (PBS). Activation was quantified by the total MFI of CD11b expression (**H**). Data are expressed as means ± SEM. *p < 0.05.

To evaluate the effect of the sEH-inhibitor TPPU on inflammation in burn injury, we conducted a 28% total body surface area (TBSA) burn injury model and evaluated the effects of TPPU administration. Previous studies revealed a beneficial effect of sEH-inhibitors in multiple inflammatory diseases and injuries^12,25–27^. The enzyme sEH metabolizes EETs to DHETs, and TPPU inhibits this process. We hypothesized that the administration of TPPU would systemically increase 14,15-EpETrE levels and decrease 14,15-DHET levels post-burn. We also hypothesized that a beneficial clinical effect can be seen reflected by the decrease of systemic IL-6 levels and that the product 14,15-DHET impairs neutrophil functionality. After 6 and 24 h post-burn injury, we saw a TPPU-dependent systemic increase of 14,15-EpETrE levels and decrease 14,15-DHET (Fig. 1A–F). All oxylipin levels are provided in the supplemental material (Suppl. Table 1 and 2). Moreover, systemic IL-6 levels were significantly decreased 6 h after burn injury and TPPU administration (Fig. 1G). When 14,15-DHET was administered after burn injury in a pilot study, we did not detect increased systemic IL-6 levels (Suppl. Figure 1A). However, the same intervention significantly decreased neutrophil activation, reflected by their total CD11b expression, 24 h post injury (Fig. 1H). Additionally, we discovered in vitro, that the CD62L-/ICAM-1- and CD62L-/ICAM-1 + phenotypes express the highest CD11b levels (Suppl. Figure 1B) and that 14,15-DHET decreases CD11b also in vitro after at least 4 h up until 24 h (Suppl. Figure 1C). The 14,15-DHET concentrations used for incubation did not affect viability of the neutrophils (Suppl. Figure 2). The results suggest that TPPU effectively inhibits sEH function resulting in beneficial prevention of excessive inflammation, as indicated by IL-6 levels, and reducing 14,15-DHET levels and neutrophil activation.

### DHET functionally impairs neutrophil Reactive Oxygen Species (ROS) production by transcriptionally impairing the NADPH oxidase complex

**Figure 2:**
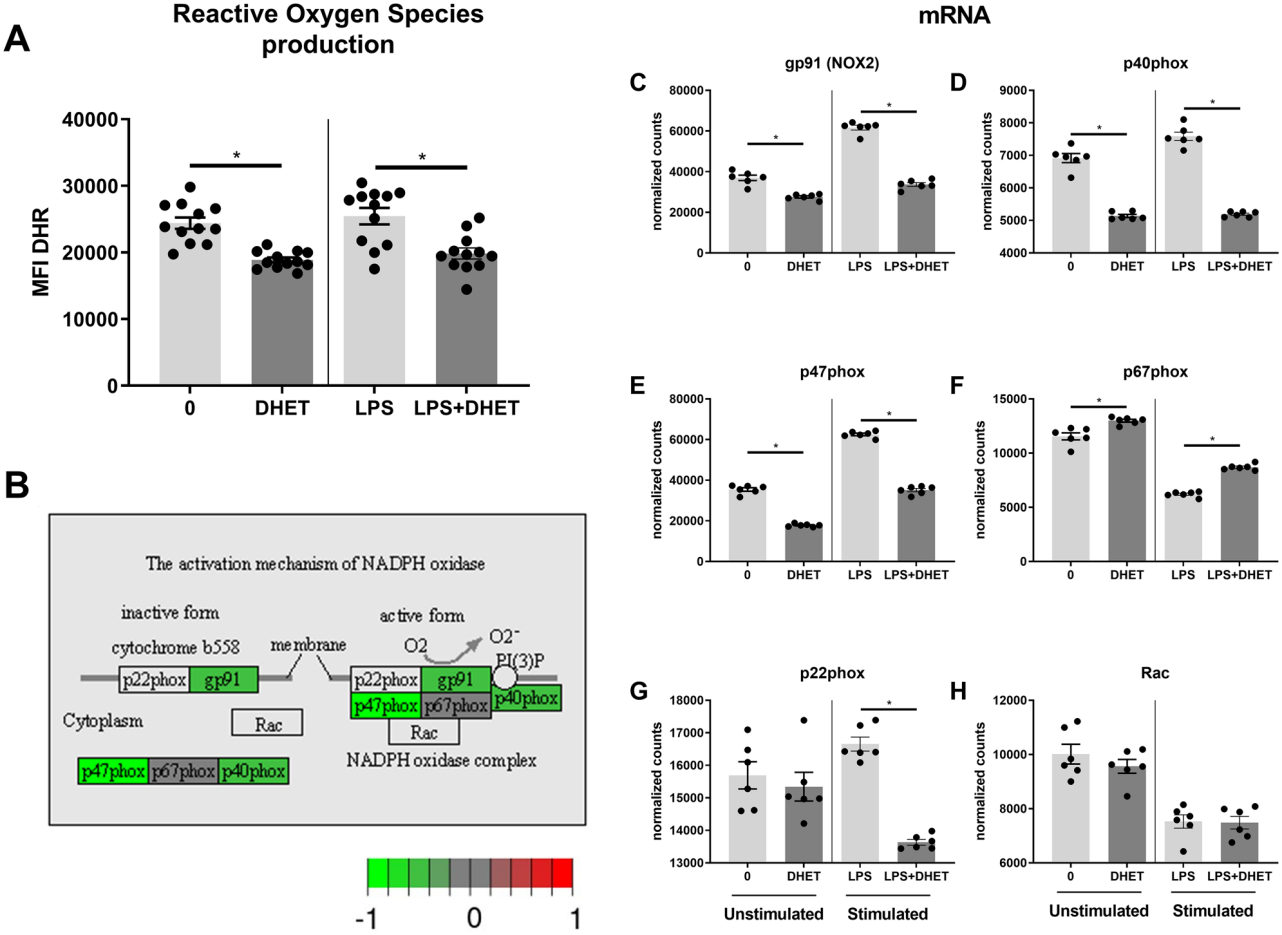
DHET functionally impairs neutrophil Reactive Oxygen Species (ROS) production in stimulated and unstimulated condition by transcriptionally impairing the NADPH oxidase complex. DHET functionally impairs neutrophil ROS production by reducing the mRNA production of key elements of the NADPH oxidase complex. Bone marrow from female C57Bl/6 mice was harvested (n = 18/ group) and incubated with 14,15-DHET followed by dihydrorhodamine (DHR) with or without lipopolysaccharide (LPS). Afterwards cells were harvested and labelled using flow cytometry. Neutrophils were identified as Ly6G positive cells. DHR passively diffuses into the cell and becomes oxidized by ROS to highly fluorescent rhodamine 123, an indicator for ROS production. The MFI of rhodamine 123 was assessed using flow cytometry (**A**). Neutrophils from bone marrow of female C57Bl/6 mice were isolated (n = 6/group). They then were incubated with 5 μM DHET or 100 ng of LPS for 3 h. mRNA sequencing was conducted. mRNA levels coding for gp91 (NOX2), p40phox, p47phox, p67phox, p22phox, Rac, were assessed (**B**–**H**). Data shown in the activation mechanism of NADPH oxidase (**B**) shows the changes of untreated neutrophils versus DHET treated neutrophils, without the addition of LPS. Data are expressed as means ± SEM. *p < 0.05. (**A**, **C**–**H**). Data are expressed in gray as *p* adjusted > 0.01. Green: log fold change > -1 (downregulated in DHET). Red: log fold change > 1. (upregulated in DHET) (**B**). The figure contains graphical elements of the Kyoto Encyclopedia of Genes and Genomes (KEGG) Pathways from the Kanehisa Laboratories. Written permission for usage in this publication was obtained from Kanehisa Laboratories on March 17, 2021.

To further assess if 14,15-DHET also blunts other neutrophil functions, we examined its effect on other key neutrophil functions for pathogen killing^28^. We hypothesized that neutrophil ROS production would be functionally impaired by 14,15-DHET and that this would be maintained by transcriptionally reducing the function of the nicotinamide adenine dinucleotide phosphate (NADPH) oxidase complex. In vitro incubation of unstimulated, as well as lipopolysaccharide (LPS)-stimulated bone marrow with 14,15-DHET did decrease neutrophil ROS production (Fig. 2A). We then analyzed expression of genes encoding the enzymes involved in NADPH oxidase (NOX) complex activation (illustrated in Fig. 2B, comparing unstimulated neutrophils with or without incubating them with DHET). On a transcriptional level, mRNA levels for three enzymes in this pathway, including gp91 (NOX2), p40phox and p47phox decreased in neutrophils treated with 14,15-DHET (Fig. 2C–E) under unstimulated conditions, whereas under LPS stimulation the same molecules plus p22phox (G) were found decreased. Under unstimulated conditions two other genes involved in this pathway, p22phox and Rac, were slightly but not significantly reduced (Fig. 2G,H). We concluded that 14,15-DHET hampers neutrophil ROS production likely in a transcriptional manner.

### DHET decreases Reactive Oxygen Species (ROS) production in mature neutrophils in a p38 and PI3K independent manner

**Figure 3:**
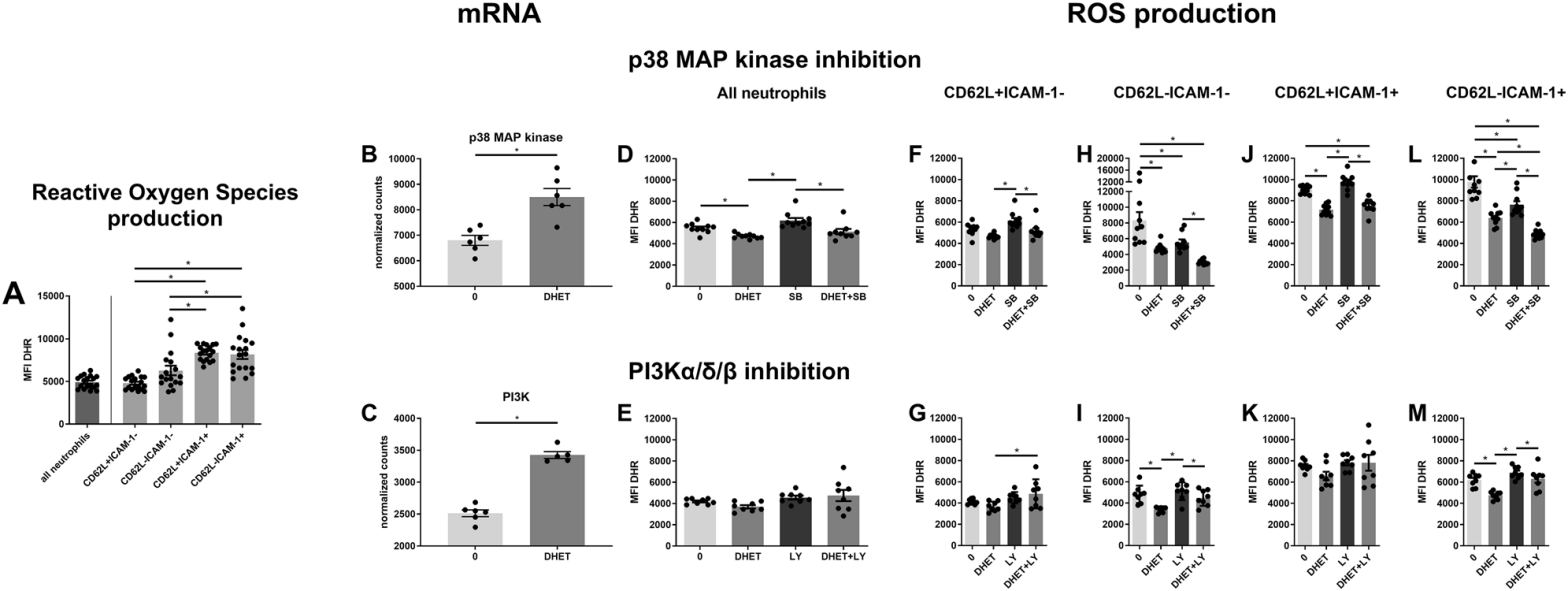
DHET decreases ROS production in mature neutrophils in an p38 and PI3K independent manner. ROS production is most potently exerted by mature (CD62L-/ + ICAM-1 +) neutrophil subtypes whereas DHET functionally decreases it. DHET increases mRNA transcription of p38 MAPK and PI3K significantly, but their blockage does not change ROS production impaired by DHET. Bone marrow from female C57Bl/6 mice was harvested (n = 18/group) and incubated with SB239063 (p38 MAP kinase inhibitor) or LY294002 (PI3Kα/δ/β inhibitor) before adding 14,15-DHET followed by dihydrorhodamine (DHR). The cells were then harvested, labeled, and analyzed using flow cytometry. Neutrophils were identified as Ly6G positive cells and subtypes identified using CD62L and ICAM-1. The MFI of the oxidized DHR was used as indicator for ROS production via flow cytometry (**A**, **D**–**M**). mRNA sequencing was conducted on isolated neutrophil cells of female C57Bl/6 mice (n = 6/group) after being incubated with 5 μM DHET for 3 h. The mRNA levels of p38MAPK and PI3K were evaluated (**B**, **C**). Data are expressed as means ± SEM. *p < 0.05.

After revealing the impairment of neutrophil ROS production, we examined which neutrophil subtypes are mainly responsible for ROS production. Moreover, we assessed whether 14,15-DHET impairs ROS production transcriptionally via p38MAPK or PI3K, both of which are known to modulate neutrophil ROS production^29,30^. We hypothesized that 14,15-DHET would also functionally alter the signaling pathways of p38MAP kinase and PI3K, and the impairment of ROS production can be reversed when these pathways are blocked. We found that neutrophil ROS production is mainly maintained by the mature CD62L + /ICAM-1 + and CD62L-/ICAM-1 + phenotype (Fig. 3A). At the gene expression level, incubation with 14,15-DHET increased p38MAP kinase and PI3K mRNA transcription (Fig. 3B,C). Interestingly, the functional inhibition of PI3K did not modulate neutrophil ROS production overall or in any of the subtypes (0 vs LY) (Fig. 3E,G,I,K,M). Blockage of p38MAPK showed a trend to increase ROS production in CD62L + ICAM-1- and CD62L + ICAM-1 + neutrophils, however not statistically significantly (0 vs SB) (Fig. 3F,J), but did decrease ROS production in the CD62L-ICAM-1- and CD62L-ICAM-1 + (0 vs SB) populations significantly (Fig. 3H,L). To assess the effect of 14,15-DHET on p38MAPK/PI3K-mediated ROS production we hypothesized that blockage of p38MAPK or PI3K might restore ROS production in 14,15-DHET treated neutrophils. However, the addition of the specific blockers did not change in ROS production in the 14,15-DHET treated cells (Fig. 3D-M). The gating strategy is displayed in Suppl. Figure 3. These results suggest that the impairment of neutrophil ROS production by 14,15-DHET is independent from p38MAP kinase and PI3K signaling pathways.

### DHET affects neutrophil functionality as it impairs the acidification of cell compartments, but does not change the phagocytic capacity, except for the CD62L-/ICAM-1-subtype

**Figure 4:**
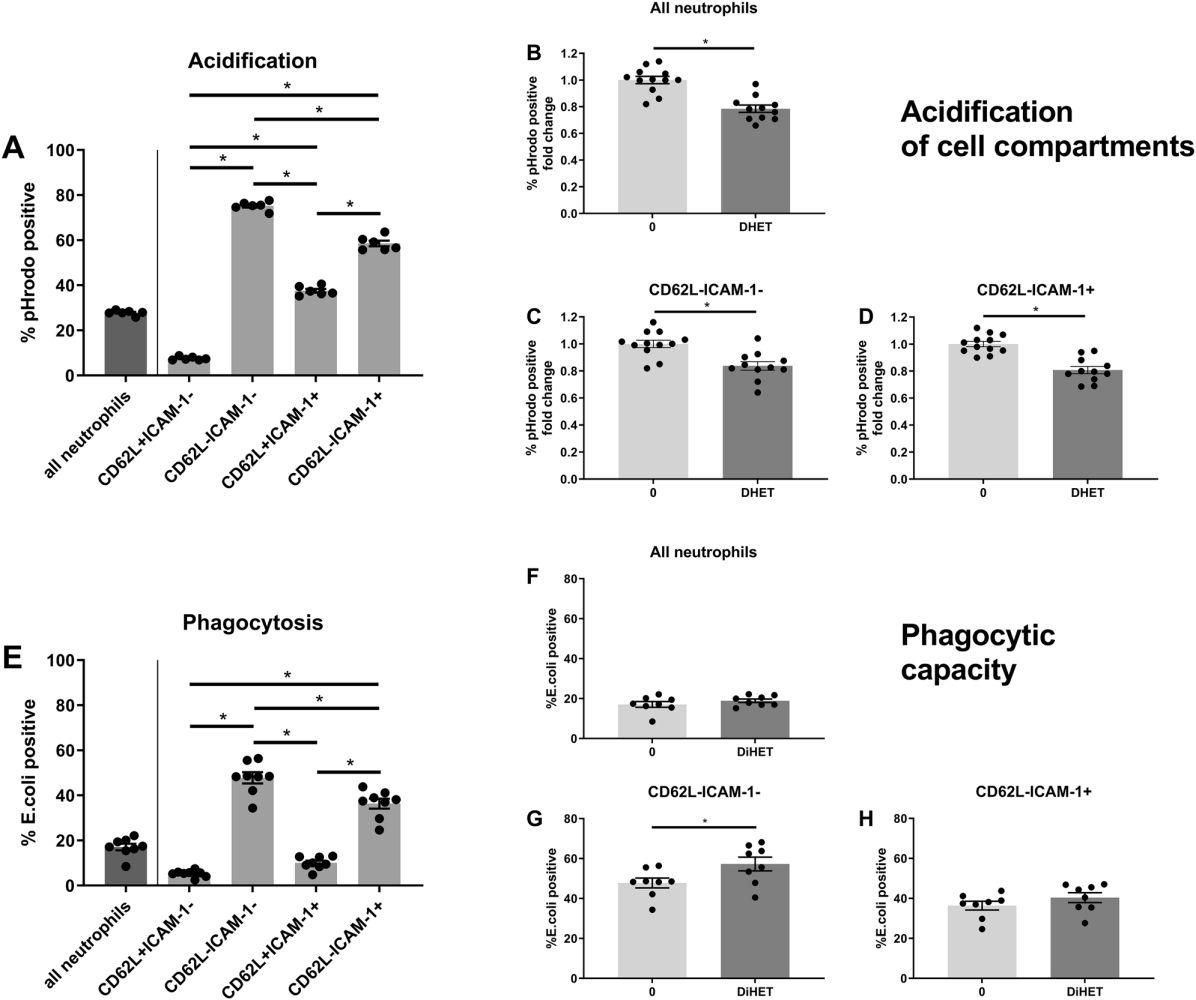
DHET affects neutrophil functionality as it impairs the acidification of cell compartments, but does not change the phagocytic capacity, except for the CD62L-/ICAM-1-subtype. The acidification in neutrophil internal cell compartments such as phagosomes and lysosomes is highest in the CD62L-/ICAM-1- and mature CD62L-/ICAM-1 + neutrophil subtypes and is significantly impaired in these subtypes by DHET. The phagocytic capacity of neutrophils was unchanged by DHET, except for the CD62L-/ICAM-1-subtype, in which it was increased. A neutrophil acidification assay was performed using pHrodo dye which fluoresces in the acidic environment of the internal cell compartments such as phagosomes and lysosomes. Bone marrow from female C57Bl/6 mice was used (n = 12). First, cells were incubated with 14,15-DHET, followed by pHrodo opsonized particles. Subsequently they were fixed and labelled for flow cytometry. Neutrophils were identified as Ly6G positive cells. CD62L and ICAM-1 were used to differentiate subtypes and the percentage of pHrodo positive cells was assessed (**A**–**D**). The phagocytic capacity of neutrophils was evaluated using opsonized *Escherichia coli* particles labelled with a fluorescent dye. Bone marrow from female C57Bl/6 mice was used (n = 8). The cells were incubated with 14,15-DHET and *E. coli* particles and consecutively labeled. The percentage of *E. coli* positive cells was measured to assess the phagocytic capacity of neutrophils and their subtypes (**E**–**H**). Data are expressed as means ± SEM. *p < 0.05.

Beside ROS production, the ability to phagocytize and digest pathogens via phagosomes and lysosomes is key for pathogen clearance^28^. We therefore hypothesized that 14,15-DHET might impair acidification of these cell compartments and the ability to phagocytize cells. We examined which neutrophil subtypes are capable of acidification and phagocytosis, and were able to attribute these functions mainly to CD62L-/ICAM-1- and CD62L-/ICAM-1 + phenotypes (Fig. 4A,E). Moreover, the acidification of cell compartments such as phagosomes and lysosomes were significantly diminished in all neutrophils and the aforementioned phenotypes after 14,15-DHET incubation (Fig. 4B–D). The phagocytic capacities in the same cells, however, were unchanged (Fig. 4F,H), or in the case of the CD62L-/ICAM-1-subtype even slightly increased (Fig. 4G). The gating strategy is displayed in Suppl. Figure 3. Mechanistically, the acidification of cell compartments is less important for neutrophils compared with macrophages^19,31^. However, neutrophil bacterial containment was found to be associated with acidification of the phagolysosome^32^. Therefore, the data suggest that 14,15-DHET impairs neutrophil cell compartment acidification while mostly not changing neutrophil phagocytosis.

### DHET impairs neutrophil migration in vitro in a CXCR1 and CXCR2 dependent manner, and reduces CXCR1 and CXCR2 expression in vivo

**Figure 5:**
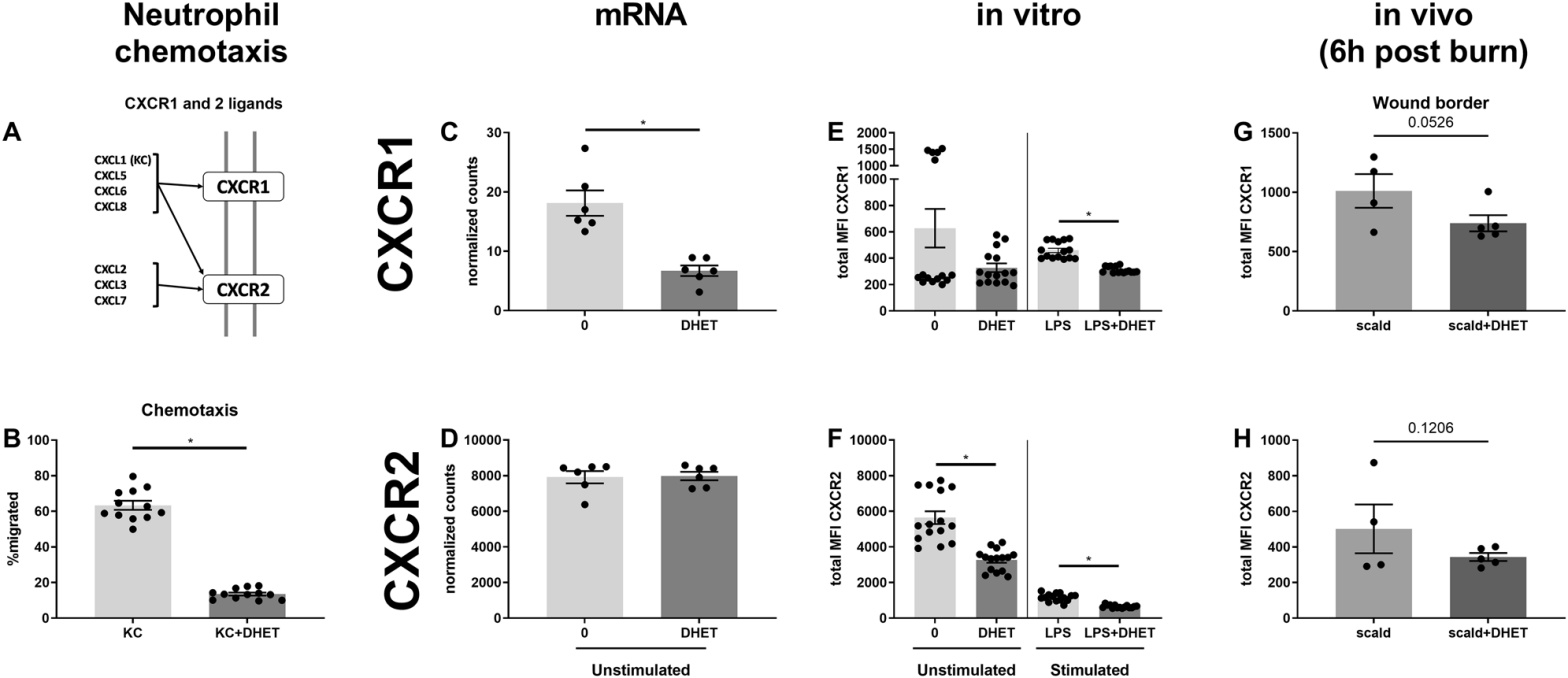
DHET impairs neutrophil migration in vitro in a CXCR1 and CXCR2 dependent way, and reduces their expression in an in vivo burn model. DHET impairs neutrophil expression of CXCR1 and 2 in vitro and in vivo, to which the strong chemoattractant chemokine (C-X-C motif) ligand 1 (CXCL1, or keratinocyte-derived chemokine: KC) binds. The migratory capability is therefore shown to be impaired. The chemokine CXCL1 (KC) is a strong chemoattractant for neutrophils and binds to CXCR1 and 2 (**A**). 14,15-DHET impairs Keratinocyte-derived Cytokine (KC, CXCL1) driven neutrophil migration in vitro after 3 h. We performed a neutrophil chemotaxis assay with bone marrow cells from C57Bl/6 mice (n = 12/ group). After harvesting bone marrow, cells were incubated on top of a transwell plate in 14,15-DHET and 100 ng of CXCL1 (KC) was added. After 3 h all cells were harvested and labelled using flow cytometry. Neutrophils were identified as Ly6G positive cells. We divided the number of neutrophils from the bottom well by the number of all neutrophils to assess the percentage of migrated neutrophils (**B**). To assess whether DHET reduces CXCR1 and 2 expression under stimulated or non-stimulated conditions we performed mRNA sequencing on isolated neutrophils of female C57Bl/6 mice after being incubated with 14,15-DHET with or without LPS (n = 6/group) (**C**,**D**). Consecutively, the expression of CXCR1 and 2 was measured in vitro. Bone marrow was incubated overnight with or without 14,15-DHET and with and without LPS. The cells were then labelled and total expression of CXCR1 and 2 measured via flow cytometry (**E**,**F**). Lastly, a burn injury model was conducted, and male CD1 IGS mice were injected with 15 µg/kg body weight 14,15-DHET or PBS as control. After 6 h the mice were euthanized and the skin surrounding the wound borders was cut out and dissociated. The cell suspension was then labelled and analyzed to assess neutrophil CXCR1 and 2 expression as total MFI via flow cytometry (**G**,**H**). Data are expressed as means ± SEM. *p < 0.05. The figure contains graphical elements of the KEGG Pathways from the Kanehisa Laboratories. Written permission for usage in this publication was obtained from Kanehisa Laboratories on March 17, 2021.

Lastly, the migratory behavior of neutrophils was studied. Neutrophils have to be recruited to the inflamed tissue to be able to clear pathogens and debris^28^. A potent chemokine attracting neutrophils to the inflamed tissue is KC (also called CXCL1), which binds to the receptors CXCR1 and CXCR2^33,34^. We hypothesized that neutrophil migration would be decreased by 14,15-DHET. The receptors and their ligands are illustrated in Fig. 5A. We observed that neutrophil migration is significantly impaired upon treatment with 14,15-DEHT in vitro (Fig. 5B). We observed that 14,15-DHET impairs the expression of CXCR1 (Fig. 5C) but not CXCR2 (Fig. 5D) on a transcriptional level. CXCR1 is mainly expressed on CD62L-/ICAM-1 + neutrophils (Suppl. Figure 4A) and CXCR2 in the CD62L + /ICAM-1- and the CD62L + /ICAM-1 + phenotypes (Suppl. Figure 4B). To determine whether these receptors are reduced in vitro we examined their expression in stimulated and non-stimulated bone marrow-derived cells (Fig. 5E,F). CXCR1 was reduced by 14,15-DHET in only stimulated cells, whereas CXCR2 was reduced by 14,15-DHET in both non-stimulated and stimulated cells. We then examined the expression in an in vivo burn injury model after 6 h and found that both CXCR1 and CXCR2 trended towards decreased expression (Fig. 5G,H). We concluded that 14,15-DHET impairs neutrophil chemotaxis most likely in a CXCR1 and CXCR2 dependent manner.

## Discussion

Several sEH-inhibitors have been found to potently reduce inflammation in various inflammatory models, but not trauma. For example, TPPU was proven to dampen inflammation in endotoxemia^11^, sepsis^12^ and intracerebral hemorrhage^13^. However, the effect on all innate immune cells has not been sufficiently studied. We sought to elucidate its effects in a trauma model and examine the mechanisms preventing excessive inflammation. Burn injury leads to an acute and robust upregulation of inflammation, mirrored by the upregulation of the pro-inflammatory cytokine IL-6 whose serum levels increase within hours and positively correlate with the size of the burned skin area^35^. Our rationale was to acutely assess IL-6 serum levels after sEH inhibition to evaluate dampening effects on inflammation in trauma. Consistent with previous reports^10,12^ we observed a robust increase of EETs and a decrease of DHETs with TPPU administration (Fig. 1A–F). The enzymatic pathway is nicely displayed in the work of Spector and Norris^2^. EETs were shown to exert potent anti-inflammatory properties^2,12,36^, e.g. by inhibiting the activation of the nuclear factor kappa B (NFκB)^37^. It is of note that genes coding for epoxide hydrolase are expressed in the skin^38^, however to our knowledge the local effects in burn injured skin tissue has not been studied. Systemically, we discovered that TPPU treatment decreased IL-6 levels, indicating that TPPU effectively ameliorates inflammation. This dampening effect on the release of pro-inflammatory cytokines can also be seen in sepsis^12^. We found the administration of DHET post burn not leading to changes in systemic IL-6 levels (Suppl. Figure 1A). This might suggest that the increase of EETs and not decrease in DHETs after TPPU administration in burn might be responsible for the decrease in IL-6. However, we suggest that given the wide distribution of systemic IL-6 levels seen in burn injury (Fig. 1G), a higher sample number would be needed to provide a clear answer to the question if DHETs significantly affect systemic IL-6 levels.

Previous sepsis and endotoxemia models only studied the anti-inflammatory effects of sEH-inhibitors and 14,15-EET^11,12^. In contrast, DHETs are less studied which led us to examine if they are responsible for detrimental effects driving inflammation. We found TPPU to decrease systemic 14,15-DHET levels, leading us to investigate whether 14,15-DHET directly effects immune function. We focused on the effect on neutrophils, as they are the first immune cells to be recruited to the injury site and quantitatively and functionally key to clear the wound of debris and potential pathogens^18^. Although our data revealed systemic 14,15-DHET levels ranging in single to low double-digit nmol/l levels, we estimate concentrations in murine skin might range from single to double digit μM concentrations when calculated based on data provided in previous murine^39^ studies. Significantly higher concentrations were also shown for liver and heart in vivo in chicken embryos^40^ and intracellularly in human non-cancerous and cancerous breast tissue^41^. As 14,15-DHET was revealed to effect cell function and gene expression at concentrations of 3 to 10 μmol/l^2^ we selected the dose of 5 μM 14,15-DHET as appropriate to estimate the effect on neutrophils mainly in the wounded skin tissue. A mechanistic examination of the 14,15-DHET effects revealed ROS production was impaired transcriptionally but was independent from p38MAPK and PI3K pathways. The acidification of cell compartments such as phagosomes was decreased, and the phagocytic capacities unchanged except for an increased in the CD62L-/ICAM-1-neutrophil subtype. Chemotaxis in vitro was impaired by the transcriptional reduction of CXCR1 but not CXCR2. Both were shown to be decreased by 14,15-DHET in vitro and trended towards decreased expression in vivo. We therefore conclude that the administration of sEH-inhibitors benefits the host in trauma-related inflammation by reducing the amount of bioactive DHET, which impairs neutrophilic key functions and consequently might lead to an impaired clearance of pathogens and debris.

CD11b serves as a marker for neutrophil activation^42^. Interestingly neutrophil activation was decreased after 24 h in the wound border (Fig. 1H). Past studies showed that increased CD11b expression can be a sign of excessive neutrophil activation^43^ which potentially fuels excessive inflammation. One might therefore argue that DHET decreasing CD11b expression shows it is dampening excessive inflammation. We believe that this is beneficial only for later time points, as in the acute phase of inflammation neutrophil activation is needed to maintain a proper immune response, otherwise leaving the host susceptible to infection^44,45^. Moreover, the analysis of neutrophil phenotypes using CD62L and ICAM-1 as markers for maturation^18,21–23^ revealed that a significant share of CD11b expression of the whole neutrophil population was attributed to the mature CD62L-/ICAM-1 + phenotype (Suppl. Figure 1B). In previous studies, we and others depicted that the CD62L-/ICAM-1 + phenotype can be considered mature and shows increased functionality compared to other phenotypes (Fig. 3A)^18,23^. Therefore, we interpret the observed effect of neutrophils being compromised in their activation as an impairment of proper immune activation and not a prevention of excessive inflammation.

To further harden this assumption, we examined another key neutrophil function, ROS production. ROS produced by neutrophils is released to clear pathogens and cell debris^46^ and to dissolve phagocyted pathogens and cell debris in the phagosomes^20^. 14,15-DHET impaired ROS production (Fig. 2A). Moreover, we could show that the impairment of the NADPH oxidase complex (Fig. 2B), which generates ROS^20^, is transcriptionally downregulated by 14,15-DHET under unstimulated and LSP-stimulated conditions (Fig. 2B–H).

As described above, we assessed the contribution of neutrophil subtypes to ROS production. We confirm previous results^23^ by showing that the mature CD62L-/ICAM-1 + and CD62L + /ICAM-1 + phenotypes contribute the most to ROS production (Fig. 3A). To examine what drives ROS production impairment we examined p38MAPK and PI3K expression, which were increased by 14,15-DHET (Fig. 3D,E). Both are reported to regulate ROS production in neutrophils in a stimulatory manner^29,30^ and both were shown to be downregulated by TPPU in LPS-activated macrophages^12^. We hypothesized that p38MAPK and PI3K expression would be impaired by DHET, thereby mediating the impairment of ROS production on a transcriptional and functional level. To our surprise, p38MAPK and PI3K mRNA expression were increased by 14,15-DHET (Fig. 3D,E). We then examined the post-translational, functional expression of the two enzymes by blocking p38MAPK and PI3K pathways using specific inhibitors. The blockage of PI3K did not decrease neutrophil ROS production (0 vs LY) (Fig. 3E,G,I,K,M). In contrast p38MAPK blockage did, although not significantly, increase ROS production in CD62L + ICAM-1- and CD62L + ICAM-1 + cells (0 vs SB) (Fig. 3F,J). It did decrease ROS production in the CD62L-ICAM-1- and CD62L-ICAM-1 + cells (0 vs SB) (Fig. 3H,L). Thus we presume that the stimulatory effect of p38MAPK on ROS production, which has been described^47^, might only apply to certain neutrophil subtypes. We therefore suggested that DHET treatment might restore functionality in all neutrophils or at least certain subtypes. However, no change in ROS production could be achieved by blocking p38MAPK and PI3K in the 14,15-DHET treated neutrophils (Fig. 3D–M). We subsequently conclude that, contradicting our initial hypothesis, the impairment of ROS production by 14,15-DHET is independent from p38MAPK and PI3K, both on transcriptional as well as post-transcriptional levels. This is important as it leads to the suggestion that DHETs do not modulate the high affinity leukotriene B4 receptor BLT1, of which DHET is a potential ligand^48^ and which is suggested to activate PI3K downstream^49^. However, the exact mechanisms will need to be examined in future studies. Moreover, multiple immune cell functions were described in the literature that are affected by the p38MAPK and PI3K pathways^50,51^. We suggest future studies to examine if the transcriptional increase of both enzymes affect neutrophil functionality in ways not studied in this manuscript.

Crucial for pathogen and debris clearance is the neutrophils’ ability to phagocytize and dissolve particles^46^. The neutrophil subtypes CD62L-/ICAM-1 + and mature CD62L-/ICAM-1 + consume and acidify particles more potently than other phenotypes (Fig. 4A,E). 14,15-DHET reduces the acidification of cell compartments (Fig. 4B–D). Neutrophils dissolve particles in their phagosomes mainly by ROS release into the phagosome^19,20^, in contrast to monocytes, which acidify their phagosomes by H + production utilizing the vacuolar (v)-ATPase^19,31^. Therefore, the pH in neutrophil phagosomes is neutral or weakly acidic, whereas monocyte phagosomes reach very low pH values^19^. It is of note that ROS release into neutrophil phagosomes is suggested to modulate pH-sensitive measurements^19^, potentially by ROS-dependent quenching of pHrodo^52^, so the pHrodo positivity observed in these experiments might be due to this effect. However, recent studies revealed that neutrophil bacterial containment is associated with acidification of the phagosome, measured with pHrodo-technique and suggested to be ROS independent^32^. Consequently, their ability to digest pathogens is decreased. 14,15-DHET did not show an effect on phagocytosis (Fig. 4F,H) except in CD62L-/ICAM-1-neutrophils were the phagocytic functionality was increased (Fig. 4G). This is of note as the CD62L-/ICAM-1-neutrophils were the main phenotype discovered in the burn wounds (Suppl. Figure 5B). However, we assume an overall impairment of their ability to clear pathogens and debris, as acidification and ROS release into phagolysosomes were shown to be important for pathogen clearing^52,53^. Clarification will require future investigations using infection models to compare the ability to clear pathogens quantified in colony forming units (CFUs). This is beyond the scope of this study as we were using a sterile burn model.

As shown above, neutrophil functionality at the site of inflammation, which is necessary for proper wound resolution, is impaired by DHET. An important prerequisite of neutrophils to exert these functions is the ability to migrate into the inflamed tissue. KC is a key chemoattractant for neutrophils released by inflamed tissue and is bound by CXCR1 and CXCR2 (Fig. 5A)^33,34^. CXCR1 and CXCR2 are relevant for neutrophil migration^33,34^, therefore several drugs are currently being tested clinically to impair recruitment to dampen inflammation^34^. More specifically CXCR2 seems to be crucial for neutrophil migration^54^ whereas CXCR1 modulation does not necessarily affect chemotaxis^55^. We showed that 14,15-DHET does impair KC-induced chemotaxis in vitro (Fig. 5B) and subsequently showed the depression of CXCR1 and 2 expression in vitro (Fig. 5E,F) and a trend towards decreased expression in vivo (Fig. 5G,H) with CXCR1 but not CXCR2 being decreased transcriptionally (Fig. 5C,D). We did not observe decreased neutrophil recruitment to the wound borders (Suppl. Figure 5A). A potential explanation might be increased systemic KC levels, driven by DHET, as other investigators showed a dose-dependent decrease of systemic KC when mice were treated with sEH-inhibitor^56^. Interestingly, in contrast to neutrophils, monocyte MCP-1 driven chemotaxis is improved by DHET^57^, leading to the assumption that DHET might have different chemotactic effects on different innate leukocytes and the effect may depend on the chemotactic stimulus. Exceeding their role in migration, numerous studies revealed that CXCR1 exerts pro-inflammatory functions on neutrophil inflammation^55,58,59^, e.g. impaired CXCR1 expression decreases neutrophil ROS production^55,59^. Therefore, CXCR1 and CXCR2 depression observed here might contribute to impairing neutrophil functionality aside from chemotaxis. In trauma patients, neutrophil CXCR2 expression was found to be decreased and the response of neutrophil CXCR1 and CXCR2 activation desensitized, increasing the risk for pneumonia^60^. Inhibition of sEH reducing systemic DHET levels might prevent this.

In summary, we were able to demonstrate that the sEH-inhibitor TPPU reduces inflammation in a murine burn injury model and reduces systemic levels of bioactive 14,15-DHET. We found 14,15-DHET to functionally impair neutrophil activation, ROS production, acidification, and the expression of CXCR1 and CXCR2, the latter potentially hampering migration. In addition, we were able to reveal that the impairment of ROS production and CXCR1 depression was compromised on a transcriptional level. These results allow three conclusions. First, we further mechanistically revealed the beneficial effect of sEH inhibitors by showing that they not only lead to increased anti-inflammatory EET levels but also reduces systemic levels of DHET, which has the capacity to impair innate immunity. Second, this provides a rationale to measure DHET levels in inflammation or cancer to evaluate potential neutrophil impairment. Third, these data provide the rationale to examine potential therapeutic modulation of systemic DHET levels to restore or dampen innate immune cell activation.

## Material and methods

### Animal models

Male C57Bl/6 and male outbred CD1 IGS mice were purchased from Charles River (Wilmington, MA, USA). C57Bl/6 mice were used for in vitro analyses and outbred CD1 IGS mice for the in vivo trauma model. All animal experiments were performed under protocols approved by the Institutional Animal Care and Use Committee (IACUC) of the University of Cincinnati (IACUC protocol no: 08-09-19-01) in accordance with all institutional and federal guidelines, and reporting in the manuscript follows the recommendations in the ARRIVE guidelines^61^. At the end of each experiment, euthanasia was performed using carbon dioxide overdose followed by cervical dislocation, a method consistent with commonly accepted norms of veterinary best practice and approved by the University of Cincinnati IACUC.

### Burn injury model and application of vehicle, TPPU and DHET

A burn injury model was applied as previously described^62^. Briefly, burn injury was inflicted via scald under 4.5% inhaled isoflurane in oxygen for anesthesia. The mice were shaved on the back and placed in a plastic cylinder with a cut-out exposing an area of their back equivalent to 28% of their TBSA calculated using the Meeh formula^63,64^. The exposed back was held in a 90 °C water bath for 9 s, leading to a third degree (full-thickness) burn injury. The mice were resuscitated with 1 ml of 0.9% normal saline injected intraperitoneally (i.p.) and placed on a 42 °C heating pad for three hours. Sham-treated mice underwent the same procedure except for exposure to the 90 °C water bath. Directly prior to burn injury (less than 60 s), mice were injected i.p. with 10 mg/kg body weight TPPU (Cayman Chemical, Ann Arbor, MI, USA) diluted in 100% polyethylene glycol (PEG) with an average molecular weight of 400 Da (Sigma-Aldrich, St. Louis, MO, USA), or with PEG vehicle for controls. In subsequent experiments, mice were injected i.p. with 15 µg/kg body weight 14,15-DHET (Cayman Chemical) diluted in phosphate buffered saline (PBS); untreated mice served as controls. Mice were euthanized after 6 and 24 h and samples were collected for analysis.

### Mass spectrometry

Following euthanasia at 6 or 24 h post-burn, whole blood was collected and centrifuged to gain blood plasma. An antioxidant solution was added (2 µl of 0.2% triphenylphosphine/0.2% butylated hydroxytoluene/0.1% ethylenediaminetetraacetic acid per 100 µl of plasma) and samples were frozen at −80 °C until analyzed. Lipid profiles were determined using solid phase extraction (SPE) followed by liquid chromatography-electrospray ionization/multi-stage mass spectrometry (LC–MS/MS)^65^.

### Serum cytokine levels

To assess serum cytokine levels, whole blood collected after euthanasia at 6 and 24 h was centrifuged to obtain serum. The serum was then analyzed to determine IL-6 levels using the BD Cytometric Bead Array (CBA) Mouse IL-6 Flex Set (BD Biosciences, San Jose, CA, USA) according to the manufacturer’s instructions.

### Isolation of neutrophils from burn wound borders

Neutrophil surface expression of CD11b, CD62L, ICAM-1, CXCR1 and CXCR2 from tissue resident cells in the burn wound borders was measured by cutting out the burn wound borders after euthanizing the mice after 6 and 24 h. The skin was cut with a 1 mm distance left and right of the visible transition between the third degree burn wound and healthy, non-burned skin, and placed in cell culture media, consisting of RPMI to which gentamycin, Minimum Essential Medium (MEM), sodium pyruvate, Penicillin–Streptomycin-Glutamine, L-Glutamine 200 mM and Cytiva HyClone Fetal Bovine Serum (Thermo Fisher Scientific) were added. The skin was then dissociated into a cell suspension using a gentle MACS Octo Dissociator with Heaters and the Multi Tissue Dissociation Kit 1 (both from Miltenyi Biotec, Bergisch Gladbach, Germany) following the manufacturer’s customized protocol for mouse whole skin (3 h incubation). The cell suspension was then washed and labelled for flow cytometry analysis as described below.

### Transcriptional analysis of neutrophils

For all in vitro experiments and gene expression analyses, bone marrow was harvested by flushing the tibia and femur of both legs. For the transcriptional analysis, neutrophils were then isolated. Magnetic bead sorting employing the autoMACS Pro Separator (Miltenyi Biotech) was conducted according to the manufacturer’s instructions, using Anti-Ly6G MicroBeads Ultra Pure mouse (Miltenyi Biotech). After isolation 2 × 10^6^ neutrophils were plated in the cell culture media described above. The cells were incubated at 37 °C for 3 h with either no stimulus, 100 ng lipopolysaccharide (LPS) (*Escherichia coli* 0111:B4, Sigma-Aldrich), 5 μM 14,15-DHET (Cayman Chemical), or the combination of the two. After incubation, mRNA was prepared using Qiagen RNeasy Mini Kit columns (Qiagen, Inc., Germantown, MD) and mRNA samples were shipped on dry ice to a commercial provider, GENEWIZ NGS bioinformatics solutions (South Plainfield, NJ, USA) for standard mRNA sequencing analysis (Standard RNA-Seq Data Analysis Package).

### Labeling and characterization of neutrophils using flow cytometry

For flow cytometry analysis, cells were washed and incubated with Fc-receptor blockage using CD16/CD32 (Mouse BD Fc Block) (clone 2.4G2 (RUO), BD Pharmingen) and 5% rat serum (Invitrogen, Carlsbad, CA, USA) for 10 min prior to labeling. Subsequently, the cells were incubated with labelling antibodies for 20 min, washed and analyzed on the Attune NxT Acoustic Focusing Cytometer (Thermo Fisher Scientific). The following fluorescent-labeled antibodies were used for cell labeling: Ly6G (clone: 1A-8), ICAM-1 (CD54) (clone: 3E2), CD62L (L-Selectin) (clone: MEL-14), CXCR1 (clone: U45-632), all from BD Biosciences and CD11b (clone: M1/70) and CXCR2 (clone: SA044-G4), from BioLegend (San Diego, CA, USA). Neutrophil subsets were characterized using CD62L (L-Selectin) and ICAM-1 (CD54). Neutrophils were considered immature when expressing a CD62L + /ICAM-1(CD54)-phenotype and mature when expressing CD62L + /ICAM-1(CD54) + or CD62L-/ICAM-1(CD54) + ^21,22^.

### Viability assay

To assess the viability of cells, cells were labelled with Annexin V (BD Biosciences) and propidium iodide (PI) (Fluka Chemie GmbH, Buchs, Switzerland). Bone marrow was incubated with either 0, 2, 5, or 10 μM 14,15-DHET for 24 h. Cells were then washed and resuspended in Annexin V Buffer (BD Biosciences), counted and diluted into 100 μl buffer containing 1 × 10^5^ cells. Each of these samples was incubated with 5 μl Annexin V antibody and 1 μl 100 μg/ml PI for 15 min. Afterwards, they were washed, placed on ice, labeled, and analyzed via flow cytometry as described above. Neutrophils were considered viable when found Annexin V and PI negative.

### Reactive oxygen species (ROS) assay and blocking of p38MAPK and PI3K

Bone marrow was incubated at 37 °C for 15 min with either no inhibitors (untreated controls), 10 μM SB239063 (p38 MAP kinase inhibitor), or 10 μM LY294002 (PI3Kα/δ/β inhibitor) (both from Cayman Chemical). Subsequently, 5 μM 14,15-DHET (Cayman Chemical) was added and incubated for 15 min followed by 1 mM dihydrorhodamine (DHR) for another 15 min. The cells were then harvested, labeled, and analyzed using flow cytometry. The median fluorescent intensity (MFI) of the oxidized DHR indicates ROS production.

### Acidification (pHrodo) assay

Opsonized *E. coli* particles labelled with pH-sensitive dyes (pHrodo Green *Escherichia coli* BioParticles; Thermo Fisher) were processed according to the manufacturer’s instructions and incubated with bone marrow at 37 °C, 5% CO_2_ for one hour, during which the neutrophils phagocyted these particles. After one hour phagocytosis was stopped by placing cells on ice and fixing them with 1% paraformaldehyde (PFA). For the rest of the procedure, cells were kept on ice and subsequently labelled for flow cytometry analysis as described above. The internal cell compartments such as phagosomes and lysosomes become acidified, which correlates with the increased intensity of the light signal of the pH-sensitive dyes, reflected in its MFI in flow cytometry. Cells that did acidify the phagocyted particles were identified as pHrodo positive cells.

### Phagocytosis

*E. coli* particles (Escherichia coli BioParticles Opsonizing Reagent; Thermo Fisher Scientific) were opsonized, washed, and incubated at 37 °C for 15 min with 1 × 10^6^ cells, according to the manufacturer’s instructions. Afterwards, cells were fixed by adding 500 μl 1% PFA for 5 min at 37 °C, washed, and labelled for flow cytometry analysis as described above. The phagocytic uptake of particles of *E. coli* was then detected using flow cytometry.

### Chemotaxis

Harvested bone marrow cells were seeded on a transwell plate (Thermo Fisher Scientific) of 3 μm pore size and incubated with 5 μM 14,15-DHET (Cayman Chemical) at 37 °C for 30 min before adding 100 ng of chemokine (C-X-C motif) ligand 1 (CXCL1, also known as keratinocyte-derived chemokine (KC)). After 3 h all cells were harvested and labeled for flow cytometry analysis. The number of neutrophils that migrated to the bottom well was divided by the number of all neutrophils to assess the percentage of migrated neutrophils.

### LPS stimulation of neutrophils in cell culture

The femur and tibia were flushed to harvest bone marrow. Two million cells of the suspension were plated per well, stimulated with 100 ng LPS (*Escherichia coli* 0111:B4, Sigma-Aldrich) and incubated at 37 °C at 5% CO_2_ for 24 h, before being labeled and analyzed via flow cytometry.

### Statistical analyses

The statistical analysis was performed with GraphPad Prism 9.0 (GraphPad Software, La Jolla, CA; graphpad.com). Outliers were identified and removed using the ROUT method (Q = 1%). All groups were tested for normality with the Shapiro–Wilk and the D`Agostino and Pearson normality test. If the groups were normally distributed, a two tailed Student’s t test comparison of two groups or one-way ANOVA with Tukey post-hoc analysis for comparisons of more than two groups was applied. A one tailed Student’s t test was used to compare CXCR1 and CXCR2 in vivo expression of neutrophils after assessing a decrease in vitro. If groups were not normally distributed, a Mann–Whitney test to compare two groups or Kruskal–Wallis test with Dunn`s multiple-comparison test analysis for comparisons of more than two groups was applied. For all in vitro experiments that included LPS-stimulation, we only compared the differences after DHET-treatment within the stimulated and non-stimulated cohorts. The results are visualized in bars with the mean ± standard error of the mean (SEM). A *p*-value of ≤ 0.05 was considered statistically significant. For the evaluation of the mRNA sequencing data an additional differential expression analysis was performed using DEseq2 method^66^. Significant results (Benjamini–Hochberg adjusted *P* value < 0.1) of KEGG pathway graphs were rendered using Pathview^67,68^.

## Supplementary Information

Supplementary Information.

## Data Availability

All data described are contained within the manuscript. Additional data not discussed here are available from the corresponding author upon reasonable request.

## References

[1] Yang T (2013). The role of 14,15-dihydroxyeicosatrienoic acid levels in inflammation and its relationship to lipoproteins. Lipids Health Dis..

[2] Spector AA, Norris AW (2007). Action of epoxyeicosatrienoic acids on cellular function. Am. J. Physiol. Cell Physiol..

[3] Morisseau C, Hammock BD (2005). Epoxide hydrolases: mechanisms, inhibitor designs, and biological roles. Annu. Rev. Pharmacol. Toxicol..

[4] Antcliffe DB (2018). Profiling inflammatory markers in patients with pneumonia on intensive care. Sci. Rep..

[5] Hamaguchi M (2019). A case series of the dynamics of lipid mediators in patients with sepsis. Acute Med. Surg..

[6] Wolfer AM (2017). Longitudinal analysis of serum oxylipin profile as a novel descriptor of the inflammatory response to surgery. J. Transl. Med..

[7] Hercule HC (2009). Interaction between P450 eicosanoids and nitric oxide in the control of arterial tone in mice. Arterioscler. Thromb. Vasc. Biol..

[8] Thomson SJ, Askari A, Bishop-Bailey D (2012). Anti-inflammatory effects of epoxyeicosatrienoic acids. Int. J. Vasc. Med..

[9] Edin ML (2018). Epoxide hydrolase 1 (EPHX1) hydrolyzes epoxyeicosanoids and impairs cardiac recovery after ischemia. J. Biol. Chem..

[10] Liu JY (2013). Substituted phenyl groups improve the pharmacokinetic profile and anti-inflammatory effect of urea-based soluble epoxide hydrolase inhibitors in murine models. Eur. J. Pharm. Sci..

[11] Zhou Y (2017). Soluble epoxide hydrolase inhibitor attenuates lipopolysaccharide-induced acute lung injury and improves survival in mice. Shock.

[12] Chen Z (2020). sEH inhibitor Tppu ameliorates cecal ligation and puncture-induced sepsis by regulating macrophage functions. Shock.

[13] Wu CH (2017). Genetic deletion or pharmacological inhibition of soluble epoxide hydrolase reduces brain damage and attenuates neuroinflammation after intracerebral hemorrhage. J. Neuroinflam..

[14] Jeschke MG (2020). Burn injury. Nat. Rev. Dis. Primers.

[15] Mulder PPG (2020). Persistent systemic inflammation in patients with severe burn injury is accompanied by influx of immature neutrophils and shifts in T cell subsets and cytokine profiles. Front. Immunol..

[16] Hanschen M, Tajima G, O'Leary F, Ikeda K, Lederer JA (2011). Injury induces early activation of T-cell receptor signaling pathways in CD4+ regulatory T cells. Shock.

[17] Beckmann N (2020). Scald Injury-induced T cell dysfunction can be mitigated by Gr1(+) cell depletion and blockage of CD47/CD172a signaling. Front. Immunol..

[18] Mortaz E, Alipoor SD, Adcock IM, Mumby S, Koenderman L (2018). Update on neutrophil function in severe inflammation. Front. Immunol..

[19] Nordenfelt P, Tapper H (2011). Phagosome dynamics during phagocytosis by neutrophils. J. Leukoc. Biol..

[20] Roos D, van Bruggen R, Meischl C (2003). Oxidative killing of microbes by neutrophils. Microbes Infect..

[21] Woodfin A (2016). ICAM-1-expressing neutrophils exhibit enhanced effector functions in murine models of endotoxemia. Blood.

[22] Ivetic A, Hoskins Green HL, Hart SJ (2019). L-selectin: A major regulator of leukocyte adhesion, migration and signaling. Front. Immunol..

[23] Sengupta S, Caldwell CC, Nomellini V (2020). distinct neutrophil populations in the spleen during PICS. Front. Immunol..

[24] Dong L (2017). Soluble epoxide hydrolase inhibitor suppresses the expression of triggering receptor expressed on myeloid cells-1 by inhibiting NF-kB activation in murine macrophage. Inflammation.

[25] Chen X (2016). Soluble epoxide hydrolase inhibition provides multi-target therapeutic effects in rats after spinal cord injury. Mol. Neurobiol..

[26] Kim J, Imig JD, Yang J, Hammock BD, Padanilam BJ (2014). Inhibition of soluble epoxide hydrolase prevents renal interstitial fibrosis and inflammation. Am. J. Physiol. Renal Physiol..

[27] Tao W, Li PS, Yang LQ, Ma YB (2016). Effects of a soluble epoxide hydrolase inhibitor on lipopolysaccharide-induced acute lung injury in mice. PLoS ONE.

[28] Mayadas TN, Cullere X, Lowell CA (2014). The multifaceted functions of neutrophils. Annu. Rev. Pathol..

[29] Kulkarni S (2011). PI3Kbeta plays a critical role in neutrophil activation by immune complexes. Sci. Signal..

[30] Qian F (2009). A non-redundant role for MKP5 in limiting ROS production and preventing LPS-induced vascular injury. EMBO J..

[31] Brisseau GF (1996). Interleukin-1 increases vacuolar-type H+-ATPase activity in murine peritoneal macrophages. J. Biol. Chem..

[32] Leliefeld PHC (2018). Differential antibacterial control by neutrophil subsets. Blood Adv..

[33] Vieira SM (2009). A crucial role for TNF-alpha in mediating neutrophil influx induced by endogenously generated or exogenous chemokines, KC/CXCL1 and LIX/CXCL5. Br. J. Pharmacol..

[34] de Oliveira S, Rosowski EE, Huttenlocher A (2016). Neutrophil migration in infection and wound repair: Going forward in reverse. Nat. Rev. Immunol..

[35] Agay D (2008). Interleukin-6, TNF-alpha and interleukin-1 beta levels in blood and tissue in severely burned rats. Eur. Cytokine Netw..

[36] Samokhvalov V (2014). PPARgamma signaling is required for mediating EETs protective effects in neonatal cardiomyocytes exposed to LPS. Front. Pharmacol..

[37] Node K (1999). Anti-inflammatory properties of cytochrome P450 epoxygenase-derived eicosanoids. Science.

[38] Hoopes SL (2017). Generation and characterization of epoxide hydrolase 3 (EPHX3)-deficient mice. PLoS ONE.

[39] Zhang Q (2018). Anti-versus pro-inflammatory metabololipidome upon cupping treatment. Cell Physiol. Biochem..

[40] Diani-Moore S, Ma Y, Gross SS, Rifkind AB (2014). Increases in levels of epoxyeicosatrienoic and dihydroxyeicosatrienoic acids (EETs and DHETs) in liver and heart in vivo by 2,3,7,8-tetrachlorodibenzo-p-dioxin (TCDD) and in hepatic EET:DHET ratios by cotreatment with TCDD and the soluble epoxide hydrolase inhibitor AUDA. Drug Metab. Dispos..

[41] Luo J (2018). 14, 15-EET induces breast cancer cell EMT and cisplatin resistance by up-regulating integrin alphavbeta3 and activating FAK/PI3K/AKT signaling. J. Exp. Clin. Cancer Res..

[42] Zhou X (2005). LPS activation of Toll-like receptor 4 signals CD11b/CD18 expression in neutrophils. Am. J. Physiol. Lung Cell Mol. Physiol..

[43] Orr Y (2007). Conformational activation of CD11b without shedding of L-selectin on circulating human neutrophils. J. Leukoc. Biol..

[44] Solomkin JS (1990). Neutrophil disorders in burn injury: Complement, cytokines, and organ injury. J. Trauma.

[45] Beckmann N (2020). Burn injury impairs neutrophil chemotaxis through increased ceramide. Shock.

[46] Nathan C (2006). Neutrophils and immunity: Challenges and opportunities. Nat. Rev. Immunol..

[47] Belambri SA (2018). NADPH oxidase activation in neutrophils: Role of the phosphorylation of its subunits. Eur. J. Clin. Investig..

[48] Behm DJ, Ogbonna A, Wu C, Burns-Kurtis CL, Douglas SA (2009). Epoxyeicosatrienoic acids function as selective, endogenous antagonists of native thromboxane receptors: Identification of a novel mechanism of vasodilation. J. Pharmacol. Exp. Ther..

[49] Jeon WK (2015). The proinflammatory LTB4/BLT1 signal axis confers resistance to TGF-beta1-induced growth inhibition by targeting Smad3 linker region. Oncotarget.

[50] Canovas B, Nebreda AR (2021). Diversity and versatility of p38 kinase signalling in health and disease. Nat. Rev. Mol. Cell Biol..

[51] Hawkins PT, Stephens LR (2015). PI3K signalling in inflammation. Biochim. Biophys. Acta.

[52] Rybicka JM, Balce DR, Chaudhuri S, Allan ER, Yates RM (2012). Phagosomal proteolysis in dendritic cells is modulated by NADPH oxidase in a pH-independent manner. EMBO J..

[53] Karavolos MH, Horsburgh MJ, Ingham E, Foster SJ (2003). Role and regulation of the superoxide dismutases of *Staphylococcus aureus*. Microbiology (Reading).

[54] Ness TL, Hogaboam CM, Strieter RM, Kunkel SL (2003). Immunomodulatory role of CXCR2 during experimental septic peritonitis. J. Immunol..

[55] Carevic M (2016). CXCR1 regulates pulmonary anti-pseudomonas host defense. J. Innate Immun..

[56] Podolin PL (2013). In vitro and in vivo characterization of a novel soluble epoxide hydrolase inhibitor. Prostaglandins Other Lipid Mediat..

[57] Kundu S (2013). Metabolic products of soluble epoxide hydrolase are essential for monocyte chemotaxis to MCP-1 in vitro and in vivo. J. Lipid Res..

[58] Swamydas M (2016). CXCR1-mediated neutrophil degranulation and fungal killing promote Candida clearance and host survival. Sci. Transl. Med..

[59] Planaguma A (2015). Combined anti CXC receptors 1 and 2 therapy is a promising anti-inflammatory treatment for respiratory diseases by reducing neutrophil migration and activation. Pulm. Pharmacol. Ther..

[60] Tarlowe MH (2005). Prospective study of neutrophil chemokine responses in trauma patients at risk for pneumonia. Am. J. Respir. Crit. Care Med..

[61] du Sert NP (2020). Reporting animal research: Explanation and elaboration for the ARRIVE guidelines 2.0. PLoS Biol..

[62] Tschop J (2009). Differential immunological phenotypes are exhibited after scald and flame burns. Shock.

[63] Gouma E (2012). A simple procedure for estimation of total body surface area and determination of a new value of Meeh's constant in rats. Lab Anim..

[64] Dawson NJ (1967). The surface-area-body-weight relationship in mice. Aust. J. Biol. Sci..

[65] Yang J, Schmelzer K, Georgi K, Hammock BD (2009). Quantitative profiling method for oxylipin metabolome by liquid chromatography electrospray ionization tandem mass spectrometry. Anal. Chem..

[66] Love MI, Huber W, Anders S (2014). Moderated estimation of fold change and dispersion for RNA-seq data with DESeq2. Genome Biol..

[67] Luo W, Brouwer C (2013). Pathview: an R/Bioconductor package for pathway-based data integration and visualization. Bioinformatics.

[68] Kanehisa M, Goto S, Furumichi M, Tanabe M, Hirakawa M (2010). KEGG for representation and analysis of molecular networks involving diseases and drugs. Nucleic Acids Res..

