# Supplementary material for: TPPU treatment of burned mice dampens inflammation and generation of bioactive DHET which impairs neutrophil function
Source: Sci Rep. 2021 Aug 16;11:16555. doi: 10.1038/s41598-021-96014-2 (PMC8368302; doi:10.1038/s41598-021-96014-2)
Supplement: Supplementary file 1 — Supplementary Information. [file 41598_2021_96014_MOESM1_ESM.pdf]

Supplemental information:

TPPU treatment of burned mice dampens inflammation and generation of bioactive DHET which impairs neutrophil function

Christian B. Bergmann[1], Bruce D. Hammock[2], Debin Wan[2], Falk Gogolla[3], Holly Goetzman[1], Charles C. Caldwell[1], Dorothy M. Supp[4,5]

1. Division of Research, Department of Surgery, College of Medicine, University of Cincinnati, Cincinnati, OH, USA.

2. Department of Entomology, University of California, Davis, CA, USA.

3. Institute of Bioinformatics, Medical University of Innsbruck, Innsbruck, Austria.

4. Division of Plastic, Reconstructive and Hand Surgery/Burn Surgery, Department of Surgery, University of Cincinnati College of Medicine, Cincinnati, OH, USA

5. Scientific Staff, Shriners Children’s Ohio, Dayton, OH, USA

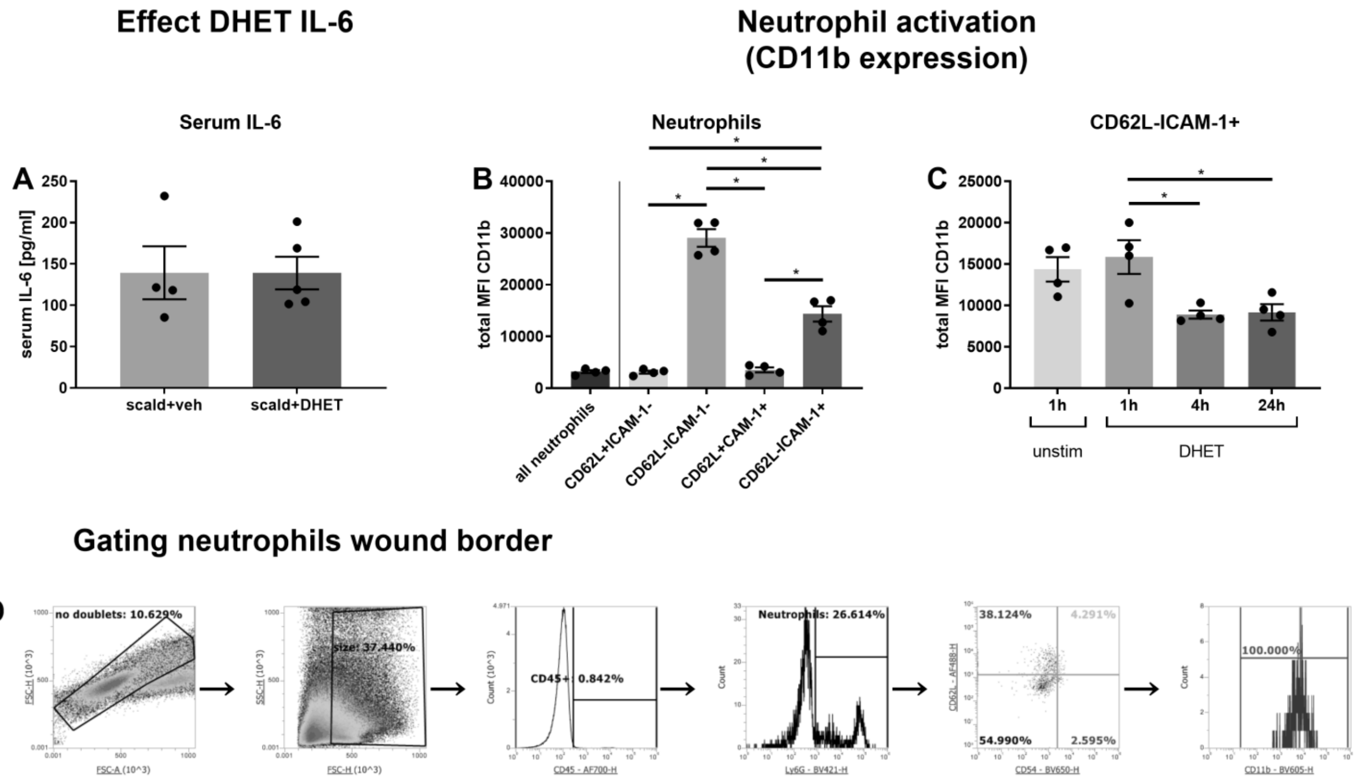

Supplemental figure 1: DHET does not change serum IL-6 levels, but impairs the activation of mature CD62L-/ICAM-1+ neutrophils

The incubation with DHET does not change systemic IL-6 levels, but decreases the expression of the activation marker CD11b in the mature CD62L-/ICAM-1+ neutrophil population. Male CD1 IGS mice were either injected with no inhibitors (untreated control) or 0.02 mg/kg 14,15-DHET in PBS was administered intraperitoneally directly post-burn or sham intervention (n=3-5/group). Injury was inflicted by a third degree burn of 28% of total body surface on the back and blood was harvested after 6h. Serum levels of IL-6 were analyzed using Cytometric Bead Array (A). Bone marrow from C57Bl/6 mice (n=4/ group) was incubated with 14,15-DHET (C) or without (B). After 1h (B,C), 4h (C) and 24h (C) the cells were harvested and labelled using flow cytometry. Neutrophils were identified as Ly6G positive cells and divided into subtypes using CD62L and ICAM-1. To assess their activation, the expression of CD11b was measured to assess differences in the MFI over time. The gating strategy used is depicted (D). Data are expressed as means ±SEM. *p<0.05.

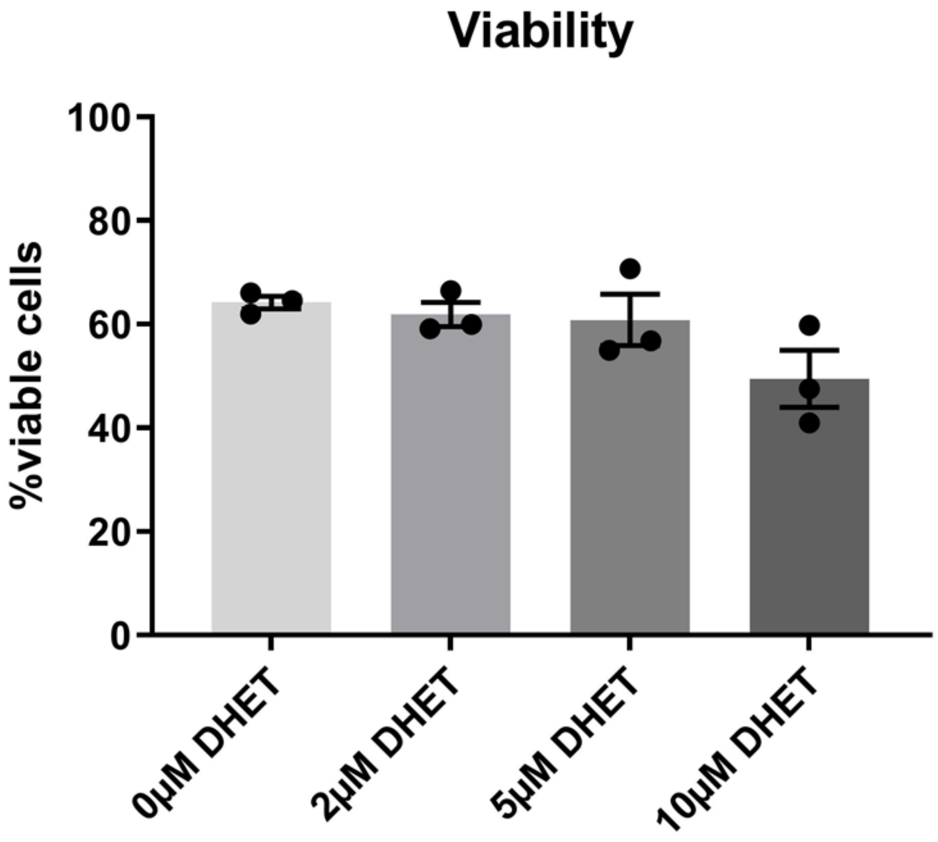

Supplemental figure 2: DHET does not affect viability of neutrophils *in vitro*

DHET does not significantly affect the viability of neutrophils *in vitro*. Bone Marrow from C57Bl/6 mice was harvested and incubated for 24 hours in 0,2,5 or 10µM 14,15-DHET cell culture solution (n=3/ group). Annexin V and propidium iodide (PI) staining was then conducted on all cells and neutrophils were identified as Ly6G positive cells using flow cytometry. Annexin V reveals cell apoptosis, and PI necrosis. Neutrophils were considered viable when determined as Annexin V and PI negative. Data are expressed as means ±SEM. $^{*}p<0.05$.

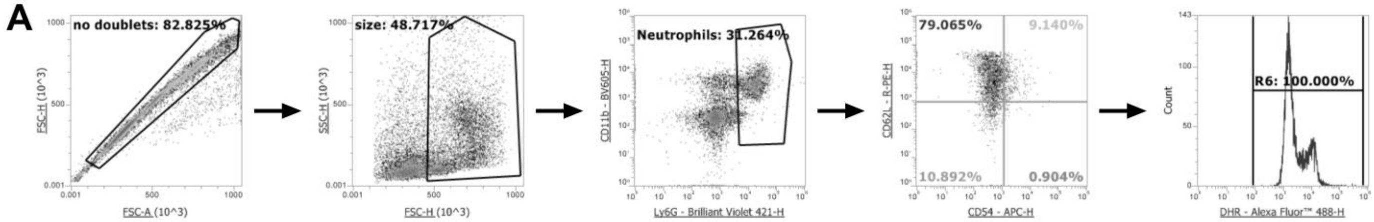

Supplemental figure 3: Gating strategy Figure 3 and 4

Neutrophils and its subtypes were identified to assess the functional changes under DHET treatment. The gating strategy for the identification of neutrophils and its subtypes using flow cytometry is displayed for Figure 3 and Figure 4.

Neutrophil CXCR1 and 2 expression

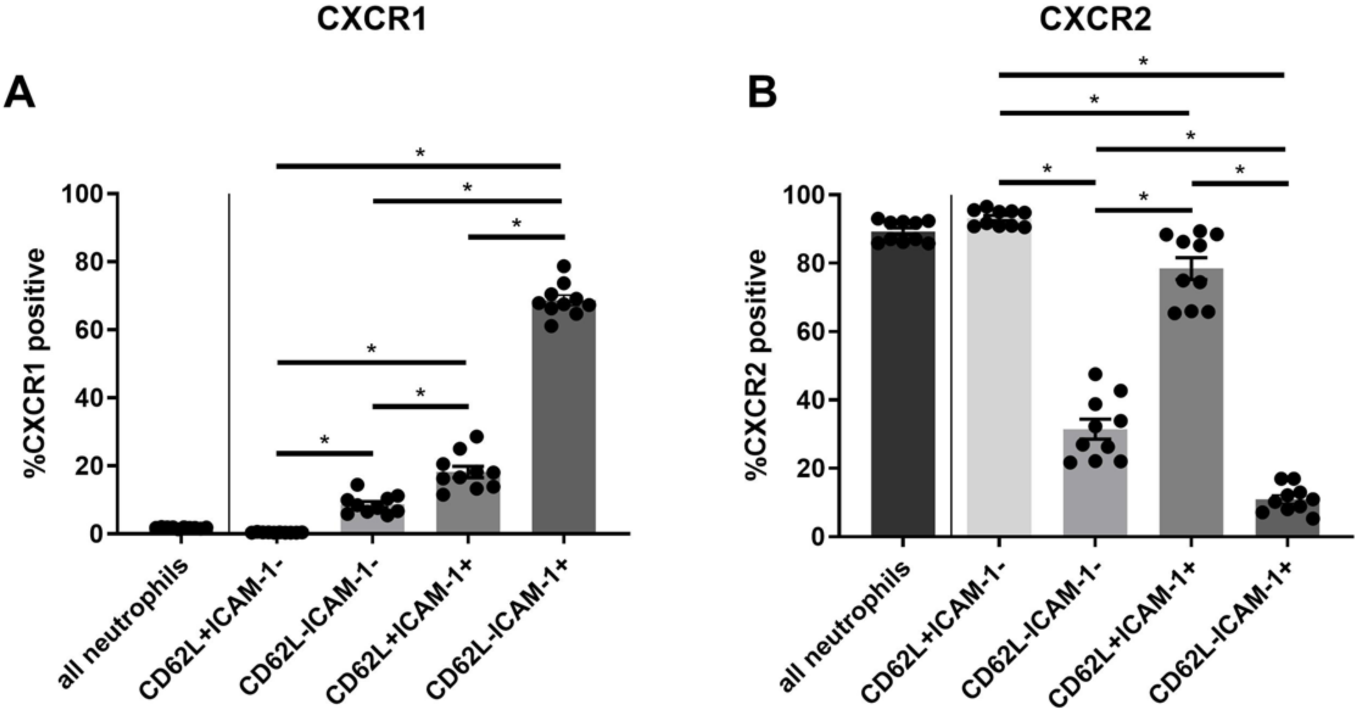

Supplemental figure 4: CXCR1 and 2 expression differs between different subtypes of bone marrow derived neutrophils

The expression of CXCR1 and 2 is highest in mature neutrophil subpopulations. Bone marrow from C57Bl/6 mice was harvested (n=10/ group). All cells were labelled using flow cytometry. Neutrophils were identified as Ly6G positive cells and divided into subtypes using CD62L and ICAM-1. Mature neutrophils were considered to express a CD62L-/ICAM-1+ phenotype. The surface expression of CXCR1 and 2 was measured (A,B). Data are expressed as means ±SEM. *$p<0.05$.

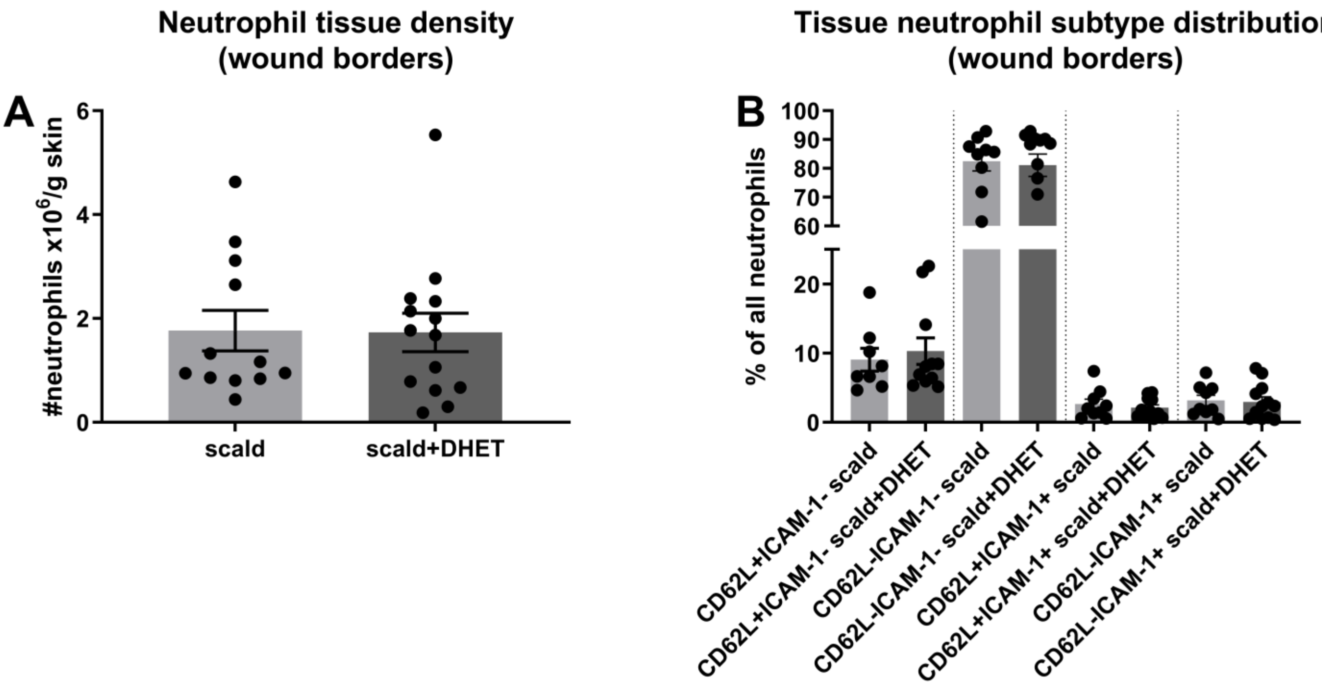

Supplemental figure 5: Chemotaxis is not impaired by 14,15-DHET *in vivo* and wound border tissue resident neutrophils are of a CD62L-/ICAM-1- phenotype

Neutrophil recruitment to the injured tissue is not impaired by 14,15-DHET and wound border tissue resident neutrophils mainly consist of the CD62L-/ICAM-1- phenotype. Male CD1 IGS mice underwent burn injury, after the injection of PBS or 15 µg/kg body weight 14,15-DHET. The skin was harvested after 6h, weighed and dissected to a cell suspension. The cells were counted on a cell counter to examine neutrophil numbers and density within the skin calculated (A). The cells were then labelled and analyzed via flow cytometry (B). Data are expressed as means ±SEM. *p<0.05.

Table S1. **Plasma oxylipin levels at 6 hr after sham treatment or scald burn in mice treated with vehicle (-) or TPPU (+). Mean values and standard deviations (SD) are listed in nmol/L. Significant p values ($p < 0.05$) are highlighted in yellow. See Table S3 for lipid definitions.**

Group:	Sham-		Sham+			Scald -			Scald+			
**Lipids**	**Mean**	**SD**	**Mean**	**SD**	**p value vs. Sham-**	**Mean**	**SD**	**p value vs. Sham-**	**Mean**	**SD**	**p value vs. Sham+**	**p value vs. Scald-**
14(15)-EpETrE	6.4	4.2	10.9	5.0	0.264	8.9	1.4	0.240	16.3	4.6	0.131	0.008
11(12)-EpETrE	4.0	2.2	5.7	2.8	0.420	6.0	0.9	0.101	10.3	4.2	0.104	0.058
8(9)-EpETrE alt	0.8	0.5	0.7	0.3	0.789	2.4	1.7	0.165	1.4	0.4	0.017	0.234
12(13)-EpOME	43.0	25.2	86.1	28.1	0.091	68.9	36.5	0.325	101.5	34.9	0.497	0.187
9(10)-EpOME	38.5	24.0	40.7	14.0	0.883	55.0	20.8	0.343	74.1	29.1	0.075	0.266
14,15-DHET (DiHETrE)	8.1	2.8	2.5	0.6	0.010	9.0	5.1	0.797	3.9	4.3	0.536	0.132
11,12-DHET (DiHETrE)	4.0	1.4	1.9	0.4	0.031	6.2	3.5	0.358	4.2	1.4	0.016	0.274
8,9-DHET (DiHETrE)	2.4	0.7	1.1	0.3	0.017	4.3	3.2	0.381	3.1	1.5	0.031	0.473
5,6-DHET (DiHETrE)	1.7	0.1	1.4	0.3	0.103	2.4	1.1	0.372	2.6	0.4	0.003	0.720
12,13-DiHOME	99.4	59.7	10.0	2.7	0.027	122.0	82.1	0.695	61.5	102.2	0.354	0.332
9,10-DiHOME	26.6	18.7	7.7	2.8	0.095	35.1	21.0	0.584	17.4	23.0	0.437	0.238
EKODE	1.6	1.3	1.1	1.2	0.621	6.1	4.6	0.152	4.0	2.4	0.064	0.372
5-HETE	5.9	1.9	5.3	1.7	0.681	8.1	1.3	0.098	8.8	4.2	0.160	0.733
6-keto-PGF1a	5.4	0.6	5.5	0.8	0.891	40.3	59.5	0.363	21.4	6.3	0.002	0.499
15-HETE	6.4	3.2	5.2	2.2	0.572	17.7	9.3	0.094	14.4	7.0	0.041	0.537
11-HETE	4.9	3.5	3.5	1.7	0.496	11.8	8.2	0.224	6.1	4.0	0.256	0.200
8-HETE	8.6	8.1	6.7	3.4	0.695	22.1	17.2	0.260	9.8	5.3	0.360	0.165
12-HETE	1671.8	2066.2	1109.1	718.3	0.626	4357.9	4226.0	0.353	1779.4	1066.3	0.320	0.222
13-HODE	125.2	23.1	100.0	38.9	0.370	206.7	81.1	0.150	172.9	64.7	0.089	0.487
9-HODE	34.8	4.9	27.8	9.4	0.302	70.3	27.0	0.071	68.9	18.5	0.005	0.925
15(S)-HETrE	5.0	2.8	3.9	1.7	0.568	9.7	6.2	0.268	6.8	2.3	0.077	0.355
15-oxo-ETE	0.7	0.2	0.7	0.5	0.922	3.1	1.7	0.061	2.8	0.6	0.001	0.715
12-oxo-ETE	436.1	679.8	228.3	207.6	0.579	1221.9	1160.6	0.335	346.8	259.5	0.483	0.138
5-oxo-ETE	0.8	0.1	0.1	0.1	0.001	0.5	0.8	0.605	1.4	2.2	0.297	0.449
13-oxo-ODE	16.5	4.0	11.6	4.5	0.197	28.1	17.9	0.322	23.4	19.2	0.274	0.699
9-oxo-ODE	43.6	35.8	23.8	17.6	0.371	66.0	24.3	0.327	72.1	38.8	0.057	0.773
15(16)-EpODE	37.5	20.1	45.0	14.9	0.591	41.9	13.0	0.716	49.5	13.6	0.650	0.391
12(13)-EpODE	2.7	1.5	4.2	1.2	0.196	3.3	1.5	0.622	4.9	2.1	0.555	0.180
9(10)-EpODE	3.9	2.5	4.1	1.5	0.896	4.5	2.1	0.722	5.6	2.8	0.349	0.481
13-HOTrE	10.5	2.8	8.4	4.4	0.510	14.9	8.2	0.418	12.9	8.0	0.347	0.717
9-HOTrE	4.3	1.0	3.3	1.7	0.422	8.1	6.4	0.366	8.3	6.4	0.178	0.961
19(20)-EpDPE	28.0	11.2	36.4	13.2	0.416	51.3	6.3	0.008	65.1	22.6	0.060	0.222
16(17)-EpDPE	6.5	2.8	8.8	3.5	0.377	14.4	5.0	0.047	23.1	11.9	0.056	0.170
13(14)-EpDPE	5.9	2.5	7.3	3.1	0.532	13.6	5.5	0.067	22.2	12.2	0.051	0.189
10(11)-EpDPE	9.4	3.5	12.7	5.5	0.401	23.0	9.7	0.063	37.5	20.2	0.050	0.184
17(18)-EpETE	5.0	1.7	7.5	2.3	0.174	6.3	3.7	0.578	9.6	2.6	0.263	0.151
14(15)-EpETE	1.7	1.0	2.4	0.6	0.337	2.0	0.8	0.642	4.2	1.5	0.061	0.021
11(12)-EpETE	1.8	1.0	2.0	0.8	0.759	2.1	0.7	0.647	3.9	2.4	0.178	0.140
8(9)-EpETE	1.1	1.1	2.0	0.6	0.199	2.4	1.1	0.143	3.3	3.3	0.466	0.581
15-HEPE	5.6	2.7	4.0	1.6	0.368	8.2	2.9	0.242	8.0	4.6	0.148	0.926
12-HEPE	11.6	1.6	12.5	9.1	0.878	6.9	7.9	0.361	13.7	8.0	0.840	0.215

8-HEPE	7.2	1.6	9.5	5.3	0.502	8.8	3.8	0.535	14.9	8.7	0.318	0.187
5-HEPE	5.0	0.8	3.9	1.1	0.200	6.0	2.1	0.481	6.6	2.8	0.113	0.690
LTB5	2.1	1.3	1.8	1.0	0.688	5.2	4.2	0.283	6.8	3.4	0.025	0.522
Resolvin E1	4.9	0.2	5.4	0.6	0.269	12.6	3.3	0.008	20.8	7.3	0.004	0.052
LXA4	0.7	0.2	0.6	0.0	0.265	1.3	0.3	0.034	2.1	0.6	0.001	0.023
15,16-DiHODE	18.5	9.2	4.7	0.7	0.027	35.9	23.5	0.276	20.4	27.7	0.301	0.367
12,13-DiHODE	3.1	1.2	0.3	0.2	0.004	3.7	2.6	0.726	2.2	3.1	0.261	0.439
9,10-DiHODE	2.6	1.7	0.6	0.3	0.059	2.8	2.1	0.896	1.3	2.6	0.605	0.343
19,20-DiHDPE	67.7	20.0	38.6	11.0	0.054	69.4	51.3	0.959	59.2	30.8	0.248	0.711
16,17-DiHDPE	18.2	2.5	8.9	3.1	0.008	10.1	5.3	0.050	6.3	2.8	0.215	0.190
13,14-DiHDPE	3.2	1.0	2.0	0.6	0.102	3.7	1.4	0.598	2.6	1.1	0.324	0.211
10,11-DiHDPE	3.1	0.2	1.3	0.2	0.000	4.4	2.0	0.327	2.9	1.7	0.116	0.238
7,8-DiHDPE	2.9	0.4	1.6	0.5	0.010	5.7	3.2	0.196	5.3	2.1	0.010	0.826
4,5-DiHDPE	3.3	0.6	7.1	2.3	0.045	8.6	1.5	0.001	20.2	5.9	0.004	0.003
17,18-DiHETE	13.4	0.7	7.0	2.7	0.011	21.1	12.3	0.334	12.0	10.7	0.396	0.247
14,15-DiHETE	2.7	0.2	1.3	0.4	0.003	3.0	2.4	0.817	0.9	1.4	0.654	0.124
11,12-DiHETE	1.3	0.3	0.7	0.2	0.029	1.6	0.8	0.607	1.0	0.5	0.312	0.207
8,9-DiHETE	1.1	0.1	0.4	0.1	0.000	1.9	1.4	0.383	0.9	1.0	0.356	0.218
5,6-DiHETE	0.0	0.0	0.0	0.0	0.343	0.1	0.0	0.128	0.1	0.1	0.029	0.097

Table S2. **Plasma oxylipin levels at 24 hr after sham treatment or scald burn in mice treated with vehicle (-) or TPPU (+). Mean values and standard deviations (SD) are listed in nmol/L. Significant p values ($p < 0.05$) are highlighted in yellow. See Table S3 for lipid definitions.**

Group:	Sham-	Sham+	Scald-	Scald+

Lipids	Mean	SD	Mean	SD	p value vs. Sham-	Mean	SD	p value vs. Sham-	Mean	SD	p value vs. Sham+	p value vs. Scald-
14(15)-EpETrE	9.4	7.6	13.0	10.6	0.605	7.1	5.2	0.550	20.9	18.5	0.448	0.077
11(12)-EpETrE	4.8	2.8	6.5	4.0	0.509	3.9	3.8	0.688	10.5	9.6	0.454	0.111
8(9)-EpETrE alt	7.4	3.3	7.4	2.0	0.984	4.3	2.1	0.083	3.5	1.5	0.002	0.367
12(13)-EpOME	129.1	22.0	256.2	84.5	0.027	82.8	53.2	0.137	296.5	188.2	0.694	0.012
9(10)-EpOME	103.1	25.9	124.9	50.2	0.469	69.1	54.6	0.278	156.3	138.7	0.675	0.141
14,15-DHET (DiHETrE)	7.5	1.5	3.0	1.6	0.007	5.9	2.6	0.305	1.6	1.1	0.105	0.001
11,12-DHET (DiHETrE)	4.1	1.2	1.9	1.1	0.032	2.9	1.2	0.147	1.5	0.8	0.465	0.016
8,9-DHET (DiHETrE)	2.0	0.4	0.9	0.3	0.005	3.0	3.1	0.548	0.8	0.7	0.729	0.053
5,6-DHET (DiHETrE)	2.7	0.7	2.2	0.8	0.358	2.4	1.3	0.683	2.2	1.3	0.962	0.652
12,13-DiHOME	107.3	34.7	18.8	4.7	0.002	132.8	76.7	0.551	20.6	16.3	0.828	0.001
9,10-DiHOME	29.2	11.5	7.4	1.1	0.009	35.3	15.3	0.513	14.1	10.4	0.238	0.005
EKODE	3.3	2.2	30.3	55.3	0.367	1.7	1.3	0.170	6.1	13.5	0.222	0.410
5-HETE	7.1	2.1	8.8	3.5	0.444	5.1	2.9	0.259	7.1	5.8	0.606	0.412
6-keto-PGF1a	18.7	5.9	16.4	7.0	0.645	18.9	18.8	0.982	17.2	11.0	0.902	0.825
15-HETE	77.4	50.3	101.0	66.2	0.592	51.2	30.0	0.298	32.7	16.8	0.011	0.139
11-HETE	60.4	35.4	67.9	54.6	0.825	35.7	20.8	0.172	22.4	10.7	0.028	0.117
8-HETE	95.3	35.7	116.3	46.7	0.502	57.9	28.2	0.085	42.4	18.9	0.001	0.207
12-HETE	17250.3	5578.9	19624.4	6108.1	0.587	9271.7	2986.8	0.012	8447.2	3635.8	0.002	0.635
13-HODE	1230.9	855.0	1258.7	974.7	0.967	668.9	347.3	0.150	453.9	187.5	0.029	0.134
9-HODE	323.6	223.8	322.2	260.1	0.993	180.0	68.7	0.138	141.2	64.7	0.065	0.266
15(S)-HETrE	91.2	63.6	138.7	95.6	0.440	38.7	28.9	0.087	23.0	11.2	0.003	0.158
15-oxo-ETE	1.7	0.8	4.4	6.2	0.428	1.6	0.9	0.863	2.0	2.9	0.359	0.724
12-oxo-ETE	6965.9	4986.0	9917.3	8074.1	0.557	8677.7	4822.3	0.589	6209.6	3760.9	0.269	0.268
5-oxo-ETE	14.6	16.2	17.5	19.7	0.828	15.0	11.5	0.966	10.8	12.3	0.461	0.495
13-oxo-ODE	161.1	107.2	129.7	83.9	0.660	81.3	38.2	0.100	52.4	21.0	0.020	0.073
9-oxo-ODE	122.6	54.7	225.7	257.2	0.462	75.6	41.9	0.142	116.8	112.0	0.295	0.375
15(16)-EpODE	71.0	15.9	83.9	35.3	0.530	42.8	7.8	0.003	86.4	50.1	0.931	0.040
12(13)-EpODE	6.8	1.4	14.8	5.1	0.023	3.8	0.8	0.002	15.6	9.4	0.880	0.006
9(10)-EpODE	9.0	2.9	11.3	5.1	0.471	4.8	2.6	0.032	12.6	8.8	0.785	0.040
13-HOTrE	77.4	53.6	57.6	42.2	0.582	39.8	19.9	0.120	25.4	9.8	0.044	0.077
9-HOTrE	10.7	3.5	11.5	2.2	0.705	6.7	1.8	0.030	6.5	3.6	0.029	0.904
19(20)-EpDPE	40.1	12.2	39.5	18.6	0.956	31.6	18.3	0.428	52.0	33.3	0.503	0.168
16(17)-EpDPE	15.0	5.3	14.9	8.9	0.983	10.8	7.0	0.326	16.7	10.8	0.779	0.232
13(14)-EpDPE	11.6	3.2	10.2	7.8	0.749	9.4	6.6	0.547	12.7	8.9	0.634	0.418
10(11)-EpDPE	15.5	4.1	15.4	10.4	0.989	12.2	9.8	0.543	19.0	14.8	0.674	0.314
17(18)-EpETE	5.7	2.1	13.4	9.8	0.175	4.3	1.4	0.234	11.1	5.3	0.594	0.005
14(15)-EpETE	2.0	0.9	4.6	2.9	0.134	1.4	0.7	0.277	4.5	2.7	0.937	0.012
11(12)-EpETE	6.0	6.0	3.4	2.1	0.447	15.0	7.3	0.067	10.3	9.8	0.201	0.308
8(9)-EpETE	2.7	1.7	2.3	2.9	0.823	2.6	1.9	0.961	3.1	3.3	0.657	0.703
15-HEPE	36.1	23.4	42.8	23.0	0.697	30.1	28.7	0.731	14.8	7.3	0.005	0.144
12-HEPE	1373.7	2034.7	402.7	545.8	0.392	4224.9	2556.5	0.090	2380.2	2867.8	0.208	0.203
8-HEPE	37.5	42.8	22.8	12.7	0.534	105.1	59.6	0.080	67.9	61.8	0.185	0.245

5-HEPE	10.9	3.4	12.1	5.8	0.722	4.2	3.5	0.013	3.3	2.2	0.002	0.541
LTB5	6.3	3.0	6.2	3.8	0.955	5.8	4.7	0.847	25.1	35.9	0.325	0.181
Resolvin E1	17.7	5.0	16.2	7.1	0.739	12.4	11.8	0.430	16.9	11.2	0.904	0.453
LXA4	1.8	0.5	1.7	0.5	0.811	1.4	1.2	0.571	3.4	3.3	0.342	0.158
15,16-DiHODE	22.7	8.5	7.5	2.3	0.014	21.4	15.5	0.883	5.7	3.4	0.346	0.010
12,13-DiHODE	4.6	1.8	1.1	0.3	0.008	4.4	1.7	0.818	0.9	0.8	0.574	0.000
9,10-DiHODE	3.4	1.2	1.1	0.5	0.011	2.9	1.5	0.597	1.0	0.9	0.899	0.008
19,20-DiHDPE	80.8	43.3	33.8	28.3	0.119	37.5	16.8	0.039	17.8	9.8	0.146	0.011
16,17-DiHDPE	16.3	8.1	7.7	4.3	0.111	7.0	1.7	0.014	3.0	1.0	0.008	0.000
13,14-DiHDPE	3.1	1.2	1.5	0.8	0.062	1.9	0.6	0.044	1.2	0.5	0.382	0.033
10,11-DiHDPE	3.6	1.4	2.0	0.8	0.091	2.6	0.9	0.183	1.7	1.1	0.660	0.097
7,8-DiHDPE	3.7	1.1	1.9	0.5	0.021	3.3	1.7	0.697	1.9	1.0	0.956	0.055
4,5-DiHDPE	3.1	1.4	7.2	3.9	0.091	3.8	4.7	0.780	11.2	14.1	0.593	0.203
17,18-DiHETE	21.0	9.4	10.0	7.1	0.114	8.3	3.7	0.010	3.0	1.0	0.011	0.001
14,15-DiHETE	4.2	1.9	2.3	1.0	0.120	1.6	0.7	0.008	0.4	0.3	0.000	0.001
11,12-DiHETE	1.6	0.7	1.0	0.3	0.170	0.8	0.4	0.039	0.6	0.3	0.079	0.203
8,9-DiHETE	0.6	0.4	0.3	0.3	0.310	0.6	0.5	0.983	0.1	0.1	0.030	0.009
5,6-DiHETE	0.1	0.1	0.1	0.0	0.739	0.1	0.1	0.145	0.1	0.1	0.808	0.201

Table S3. Alternative names, systematic names, and PubChem compound identification (CID) numbers (https://pubchem.ncbi.nlm.nih.gov/) for chemicals listed in Tables S1 and S2.

Abbreviated name	Alternative name (if available)	Systematic Name	PubChem CID
14(15)-EpETrE	14,15-EET	14,15-epoxy-5Z,8Z,11Z-eicosatrienoic acid	5283205
11(12)-EpETrE	11,12-EET	11,12-epoxy-5Z,8Z,14Z-eicosatrienoic acid	5283204
8(9)-EpETrE alt	8(9)-EpETrE-EA	N-((+/-)-8(9)-epoxy-5Z,11Z,14Z-eicosatrienoyl)-ethanolamine	16061182
12(13)-EpOME	Vernolic acid; iso-leukotoxin	(+/-)-12(13)-epoxy-9Z-octadecenoic acid	5356421
9(10)-EpOME	Coronaric acid; Leukotoxin	9,10-epoxy-12Z-octadecenoic acid	6246154
14,15-DHET (DiHETrE)	(+/-)14,15-DiHETrE	14,15-dihydroxy-5Z,8Z,11Z-eicosatrienoic acid	5283147
11,12-DHET (DiHETrE)	(+/-)11,12-DiHETrE	11,12-dihydroxy-5Z,8Z,14Z-eicosatrienoic acid	5283146
8,9-DHET (DiHETrE)	(+/-)8,9-DiHETrE	8,9-dihydroxy-5Z,11Z,14Z-eicosatrienoic acid	5283144
5,6-DHET (DiHETrE)	(+/-)5,6-DiHETrE	5,6-dihydroxy-8Z,11Z,14Z-eicosatrienoic acid	5283142
12,13-DiHOME	iso-leukotoxin diol	12,13-dihydroxy-9Z-octadecenoic acid	10236635
9,10-DiHOME	Leukotoxin diol	9,10-dihydroxy-12Z-octadecenoic acid	9966640
EKODE	trans-EKODE-(E)-Ib	9-oxo-11-(3-pentyloxiran-2-yl)undec-10-enoic acid	53394018
5-HETE	15-HETE	5-hydroxy-5Z,8Z,11Z,13E-eicosatetraenoic acid	9966861
6-keto-PGF1a	6-keto-Prostaglandin F1α	6-oxo-9S,11R,15S-trihydroxy-13E-prostenoic acid	5280888
15-HETE	15-HETE	5-hydroxy-5Z,8Z,11Z,13E-eicosatetraenoic acid	9966861
11-HETE	11-HETE	11-hydroxy-5Z,8Z,11E,14Z-eicosatetraenoic acid	14123410
8-HETE	8-HETE	8-hydroxy-5Z,9E,11Z,14Z-eicosatetraenoic acid	11976122
12-HETE	12-HETE	12-hydroxy-5Z,8Z,10E,14Z-eicosatetraenoic acid	13786989
13-HODE	13S-HODE	13S-hydroxy-9Z,11E-octadecadienoic acid	6443013
9-HODE	9-HODE	9-hydroxy-10E,12Z-octadecadienoic acid	5282944
15(S)-HETrE	15S-HETrE	15S-hydroxy-8Z,11Z,13E-eicosatrienoic acid	5283145
15-oxo-ETE	15-Oxo-ETE	15-oxo-5Z,8Z,11Z,13E-eicosatetraenoic acid	5280701
12-oxo-ETE	12-oxo-ETE	12-oxo-5Z,8Z,10E,14Z-eicosatetraenoic acid	5283162
5-oxo-ETE	5-Oxo-ETE	5-oxo-6E,8Z,11Z,14Z-eicosatetraenoic acid	5283159
13-oxo-ODE	13-KODE	13-keto-9Z,11E-octadecadienoic acid	6446027
9-oxo-ODE	9-OxoODE	9-oxo-10,12-octadecadienoic acid	5283011
15(16)-EpODE	15,16-EpODE	15,16-epoxy-13-OH-9Z,11E-octadecadienoic acid	16061056
12(13)-EpODE	α-12(13)-EpODE	12(13)-epoxy-9Z,15Z-octadecadienoic acid	16061061
9(10)-EpODE	9,10-EpODE	9S,10-epoxy-10,12Z-octadecadienoic acid	16061051
13-HOTrE	13-HoTrE	13S-hydroxy-9Z,11E,15Z-octadecatrienoic acid	16061072
9-HOTrE	9-HOTrE	9S-hydroxy-10E,12Z,15Z-octadecatrienoic acid	6439873
19(20)-EpDPE	19(20)-EpDPE	(+/-)-19(20)-epoxy-4Z,7Z,10Z,13Z,16Z-docosapentaenoic acid	11631565
16(17)-EpDPE	16(17)-EpDPE	(+/-)-16(17)-epoxy-4Z,7Z,10Z,13Z,19Z-docosapentaenoic acid	14392758
13(14)-EpDPE	13(14)-EpDPE	(+/-)-13(14)-epoxy-4Z,7Z,10Z,16Z,19Z-docosapentaenoic acid	11674605
10(11)-EpDPE	10(11)-EpDPE	(+/-)-10(11)-epoxy-4Z,7Z,13Z,16Z,19Z-docosapentaenoic acid	11638767
17(18)-EpETE	17(18)-EpETE	(+/-)-17(18)-epoxy-5Z,8Z,11Z,14Z-eicosatetraenoic acid	16061089
14(15)-EpETE	14(15)-EpETE	(+/-)-14(15)-epoxy-5Z,8Z,11Z,17Z-eicosatetraenoic acid	16061088
11(12)-EpETE	11(12)-EpETE	(+/-)-11(12)-epoxy-5Z,8Z,14Z,17Z-eicosatetraenoic acid	16061087
8(9)-EpETE	8(9)-EpETE	(+/-)-8(9)-epoxy-5Z,11Z,14Z,17Z-eicosatetraenoic acid	16061086

15-HEPE	15-HEPE	(5Z,8Z,11Z,13E,17Z)-16-hydroxyicosa-5,8,11,13,17-pentaenoic acid	53480357
12-HEPE	(+/-)-12-HEPE	(+/-)-12-hydroxy-5Z,8Z,10E,14Z,17Z-eicosapentaenoic acid	10041593
8-HEPE	(+/-)-8-HEPE	(+/-)-8-hydroxy-5Z,9E,11Z,14Z,17Z-eicosapentaenoic acid	16061128
5-HEPE	(+/-)-5-HEPE	(+/-)-5-hydroxy-6E,8Z,11Z,14Z,17Z-eicosapentaenoic acid	6439678
LTB5	Leukotriene B5	5S,12S-dihydroxy-6Z,8E,14Z,17Z-eicosapentanenoic acid	5283125
Resolvin E1	Resolvin E1	5S,12R,18R-trihydroxy-6Z,8E,10E,14Z,16E-eicosapentaenoic acid	10473088
LXA4	Lipoxin A4	5S,6R,15S-trihydroxy-7E,9E,11Z,13E-eicosatetraenoic acid	5280914
15,16-DiHODE	α-15,16-DiHODE	(+/-)-15,16-dihydroxy-9Z,12Z-octadecadienoic acid	16061068
12,13-DiHODE	α-12,13-DiHODE	(+/-)-12,13-dihydroxy-9Z,15Z-octadecadienoic acid	16061067
9,10-DiHODE	α-9,10-DiHODE	(+/-)-9,10-dihydroxy-12Z,15Z-octadecadienoic acid	16061066
19,20-DiHDPE	19,20-DiHDPA	(+/-)-19,20-dihydroxy-4Z,7Z,10Z,13Z,16Z-docosapentaenoic acid	16061148
16,17-DiHDPE	16,17-DiHDPE	(+/-)-16,17-dihydroxy-4Z,7Z,10Z,13Z,19Z-docosapentaenoic acid	16061147
13,14-DiHDPE	13,14-DiHDPE	(+/-)-13,14-dihydroxy-4Z,7Z,10Z,16Z,19Z-docosapentaenoic acid	16061146
10,11-DiHDPE	10,11-DiHDPE	(+/-)-10,11-dihydroxy-4Z,7Z,13Z,16Z,19Z-docosapentaenoic acid	16061145
7,8-DiHDPE	7,8-DiHDPE	(+/-)-7,8-dihydroxy-4Z,10Z,13Z,16Z,19Z-docosapentaenoic acid	16061144
4,5-DiHDPE	4,5-DiHDPE	4,5-Diyhdroxy-7Z,10Z,13Z,16Z,19Z-docosapentaenoic acid	14429107
17,18-DiHETE	17,18-DiHETE	(+/-)-17,18-dihydroxy-5Z,8Z,11Z,14Z-eicosatetraenoic acid	16061120
14,15-DiHETE	14,15-DiHETE	(+/-)-14,15-dihydroxy-5Z,8Z,11Z,17Z-eicosatetraenoic acid	16061119
11,12-DiHETE	11,12-DiHETE	(+/-)-11,12-dihydroxy-5Z,8Z,14Z,17Z-eicosatetraenoic acid	16061121
8,9-DiHETE	8,9-DiHETE	(+/-)-8,9-dihydroxy-5Z,11Z,14Z,17Z-eicosatetraenoic acid	16061118
5,6-DiHETE	5S,6R-DiHETE	5S,6R-dihydroxy-7E,9E,11Z,14Z-eicosatetraenoic acid	5283160

Supplemental table 1-3: TPPU administration in burn injury alters systemic oxylipin composition 6 and 24 hours post-burn

TPPU leads to significant changes in the blood serum oxylipin composition 6 and 24 hours after burn injury. TPPU, which is a soluble epoxide hydrolase (sEH) inhibitor was administered in a concentration of 10 mg/kg body weight in polyethylene glycol 400 (PEG) or PEG as control intraperitoneally directly post-burn injury or sham intervention in male CD1 IGS mice (n=3-9/group). Burn injury was induced by exposing 28% of total body surface on the back to 90°C hot water leading to a third degree burn of that area. After 6 and 24 hours blood was harvested and serum levels of oxylipins were assessed using mass spectroscopy (Table S1 and 2). The systematic names of all individual oxylipins are provided (Table S3).
